# Functional Characterization of TaFUSCA3, a B3-Superfamily Transcription Factor Gene in the Wheat

**DOI:** 10.3389/fpls.2017.01133

**Published:** 2017-06-28

**Authors:** Fusheng Sun, Xiyan Liu, Qiuhui Wei, Jiannan Liu, Tianxiang Yang, Liyang Jia, Yuesheng Wang, Guangxiao Yang, Guangyuan He

**Affiliations:** The Genetic Engineering International Cooperation Base of Chinese Ministry of Science and Technology, Key Laboratory of Molecular Biophysics of Chinese Ministry of Education, College of Life Science and Technology, Huazhong University of Science and TechnologyWuhan, China

**Keywords:** TaFUSCA3, TaSPA, seed storage protein, complementation of the loss-of-function, wheat

## Abstract

The end-use quality of wheat, including its unique rheology and viscoelastic properties, is predominantly determined by the composition and concentration of gluten proteins. While, the mechanism regulating expression of the seed storage protein (SSP) genes and other related genes in wheat remains unclear. In this study, we report on the cloning and functional identification of *TaFUSCA3*, a B3-superfamily transcription factor (TF) gene in wheat. Sequence alignment indicated that wheat and barley *FUSCA3* genes are highly conserved. Quantitative reverse-transcription (qRT)-PCR analysis showed that the transcript of *TaFUSCA3* was accumulated mostly in the stamens and the endosperms of immature wheat seeds. Yeast-one-hybrid results proved that the full-length TaFUSCA3 and its C-terminal region had transcriptional activities. Yeast-two-hybrid and bimolecular fluorescence complementation assays indicated that TaFUSCA3 could activate the expression of the high molecular weight glutenin subunit gene *Glu-1Bx7* and interact with the seed-specific bZIP protein TaSPA. DNA-protein-interaction enzyme-linked immunosorbent assay demonstrated that TaFUSCA3 specifically recognizes the RY-box of the *Glu-1Bx7* promoter region. Transient expression results showed that TaFUSCA3 could *trans*-activate the *Glu-1Bx7* promoter, which contains eight RY-box sequences. TaFUSCA3 was unable to activate the downstream transcription when the RY-box was fully mutated. TaFUSCA3 could activate the transcription of the *At2S3* gene promoter in a complementation of loss-of-function experiment using the *Arabidopsis thaliana* line *fus3-3*, which is a *FUSCA3* mutant, demonstrating the evolutionary conservation of the *TaFUSCA3* gene. In conclusion, the wheat B3-type TF, TaFUSCA3, is functional conserved between monocot and dicot, and could regulate SSP gene expression by interacting specifically with TaSPA.

## Introduction

Wheat (*Triticum aestivum* L.) is a very important cereal crop throughout the world, which can be made into diverse edible products for human beings because of the distinct viscoelastic characteristics of its cohesive dough conferred by the SSPs in the endosperm (Shewry, 2009). The HMW-GSs of SSPs play important roles in wheat gluten as the skeletal network that determines its structure and formation (Shewry and Tatham, 1990). The formation and accumulation of SSPs is controlled by various essential TFs at different developmental stages both in monocotyledons and dicotyledons (Plessis et al., 2013; González-Calle et al., 2014; Zhang et al., 2015). A group of important TFs, the AFL subfamily containing a plant-specific B3 DNA binding domain that takes part in this process, has been identified. Members of this family include ABSCISIC ACID-INSENSITIVE3 (ABI3), FUSCA3 (FUS3), LEAFY COTYLEDON1 (LEC1), and LEAFY COTYLEDON1 (LEC2), which regulate the desiccation tolerance and dormancy of the maturation stage of *Arabidopsis thaliana* (Stone et al., 2001; Swaminathan et al., 2008; Roscoe et al., 2015). The AFL subfamily genes are able to affect the biosynthesis of hormones and regulate the expression activity of downstream genes via protein–protein interactions (Carbonero et al., 2016). If any of the members including ABI3, FUS3, LEC1, and LEC2 are mutated, it will result in the reduced accumulation of endosperm-specific storage proteins. Besides, the TFs are interlocked in an intricate stratified reticular regulatory network by virtue of mutual interconnections (To et al., 2006).

The expression of SSP genes is regulated under strict organ-specific and temporal order during the seed development process (Kawagoe and Murai, 1992; Onodera et al., 2001), in which *cis*-acting elements of relevant promoters play an important role. The various accurate *cis*-acting elements, containing the B-box (5′-CAAACACC-3′), the RY-box (5′-CATGCA-3′), and the G-box (5′-CACGTG-3′) in the promoter of the gene encoding NapA, have been authenticated in *Brassica napus* (Ezcurra et al., 1999; Fauteux and Strömvik, 2009). The above-mentioned elements are functionally conserved and correlative in other species, such as the promoters of 2S albumins from *A. thaliana* (Kroj et al., 2003). Studies have revealed the significance of B3-type TFs and RY boxes (Mönke et al., 2004). FUSCA3 may directly regulate *At2S3* and *napA* by specifically recognizing the specific RY-box element in their promoters (Reidt et al., 2000). However, FUSCA3 could also indirectly control seed filling and the accumulation of seed storage reserves through an additional regulatory factor, such as TRANSPARENT TESTA GLABRA 1 (TTG1) (Chen et al., 2015), and the functional protein AtFUSCA3 with a conserved B3 domain has been shown to regulate late embryogenesis and seed maturation in *A. thaliana* (Luerssen et al., 1998). The seeds in the *FUSCA3* mutant of *A. thaliana* cannot process dormancy and they are further distinguished by the reduced accumulation of multiple storage substances and abundant production of anthocyanin (Roscoe et al., 2015).

Reserve substances consisting of starches, SSPs, and fatty acids are primarily accumulated in the endosperm of seeds in monocotyledons (Olsen and Becraft, 2013). Regulation of SSPs’ expression depends on the interactions between *cis*-acting elements and *trans*-activating TFs (Mena et al., 2002; Shewry and Halford, 2002; Juhász et al., 2011). A diverse array of *cis*-acting elements in the promoters of these proteins has been verified. The DOF recognition site (PB-box, 5′-TGTAAAG-3′), LAV recognition site (RY-box, 5′-CATGCA-3′), bZIP recognition sites (GLM-box, 5′-ATGAG/CTCAT-3′ and G-box, 5′-TTACGTGG-3′), R2R3MYB recognition site (5′-AACAAC-3′), and other essential motifs are conserved in the promoters of genes encoding cereal SSPs (Wu et al., 2000; Geng et al., 2014).

In maize, opaque2 (O2) heterodimerizing proteins (OHP) and DOF proteins (MPBF) can synergistically activate the expression of the γ-*Zeins* 27 gene encoding an alcohol soluble protein through the O2-like box (Pysh et al., 1993; Zhang et al., 2015). In barley, HvFUSCA3, binding to the RY motif of *Itr1* and *Hor2* promoters, activates the transcription of these genes by interacting with BLZ2 (Moreno-Risueno et al., 2008). In wheat, TaSPA, the O2-like bZIP factor, recognizes the GLM (GCN4-like motif) in the bi-factorial endosperm box to regulate expression of the low molecular weight glutenin subunit (LMW-GS) gene (Albani et al., 1997). TaGAMyb, which attaches to the R2R3MYB family, has been identified to interact with the histone acetyltransferase TaGCN5 to activate the expression of *TaGLU-1Dy*, a HMW-GS gene in wheat (Guo et al., 2015). Regulation of the expression of SSPs is accomplished by the integrated action of multitudinous TFs (Shekhar et al., 2016). A previous study showed that the seed-specific storage protein genes in *A. thaliana* had a distinctly lower expression level in a *FUSCA3* mutant line (Bäumlein et al., 1994).

The RY motif is conserved among the promoters of the monocot prolamin and the dicot SSP genes. Whether or not the RY motif could be directly recognized by a B3 transcriptional factor, which plays an important part in the regulation of SSP expression, and the downstream target of regulation have not been documented previously in wheat. In order to evaluate whether the FUSCA3-like homologous gene in wheat could regulate the expression of SSP genes, we cloned and characterized *TaFUSCA3* in wheat and confirmed its function in the regulation of expression of the SSP Ta1Bx7 in this study. Our results showed that TaFUSCA3 is able to bind to and activate the *Ta1Bx7* gene specifically through the RY-box in the promoter region. Moreover, it was shown that TaFUSCA3 interacts with the seed-specific bZIP protein TaSPA *in vivo*. Complementation of the loss-of-function *FUSCA3* mutant in the *A. thaliana* mutant line *fus3-3* by TaFUSCA3 resulted in SSP gene expression in seeds.

## Materials and Methods

### Plant Materials

Seeds of Bobwhite wheat (*T. aestivum* cv. Bobwhite), a model wheat cultivar, were germinated in sterile water for 1 week and then transplanted into a climate-controlled chamber (16 h light/8 h dark cycle at 26°C). Different organs including roots, young stems and young leaves were collected from young seedlings, while stamens, mature stems, mature leaves, flag leaves, endosperms, and pistils were collected from mature wheat plants for organ-specific gene expression analysis. The endosperms were harvested from the seeds at 4, 8, 12, 16, 20, 24, 28, and 32 DAP, respectively, and stored at -80°C.

### Cloning and Bioinformatic Analysis of *TaFUSCA3*

Total RNA extracted from young wheat seeds (20 DAP) was used to synthesize first-strand cDNAs and to amplify the cDNA of *TaFUSCA3*. To identify the putative *TaFUSCA3* gene in wheat, the sequence of the *HvFUSCA3* gene (GenBank No.: AM418838) from *Hordeum vulgare* was used as a query probe to blast the data library of wheat^1^ (Moreno-Risueno et al., 2008). A *T. aestivum* gene sequence with high identity to the ORF of *HvFUSCA3* was identified. The sequence analysis showed that it included a complete ORF sequence, which was detected by ORF Finder^2^. This gene was then amplified from cDNA by the use of degenerate primers (Supplementary Table S1). The PCR product was purified and ligated into pMD18-T simple vector (Takara, Dalian, China), which was sequenced to confirm its veracity.

The FUSCA3 sequences from seven plant species were searched for and compared at the NCBI^3^ and were used for phylogenetic analysis. The alignment of different sequences was performed by using the software DNAMAN, MEGA (version 5.1), and ClustalX2.0, and the neighbor-joining method was used to construct a phylogenetic tree (Saitou and Nei, 1987).

### Subcellular Localization and Bimolecular Fluorescence Complementation (BiFC) of TaFUSCA3 and TaSPA

The coding sequences of *TaFUSCA3* and *TaSPA* (excluding the termination codon) were amplified using primers described in Supplementary Table S1 for transient expression in onion epidermal cells. The PCR-amplified products were then subcloned into the pCAMBIA1303-35S-*GFP* expression vector and pSPYNE-35S/pUC-SPYNE and pSPYCE-35S/pUC-SPYCE vectors for BiFC. The experiment was performed as described by Walter et al. (2004). Information on the vectors used in our study is listed in Supplementary Table S2.

### *Trans*-activation and Binding Activity Analysis of TaFUSCA3 in Yeast

The assay for analysis of transcription activation properties of TaFUSCA3 was conducted in the *AH109* yeast strain. The complete coding sequences of *TaTAFUS3* (full-length ORF), and N-terminal (*TaFUSCA3-N*), B_3_ domain (*TaFUSCA3-B_3_*), and C-terminal (*TaFUSCA-C*) fragments were amplified by PCR using specific primers P17-P20, respectively (Supplementary Table S1).

Binding activity between FUSCA3 and the RY-box of both the *At2S3* and *Ta1Bx7* gene promoter regions was assayed by using the yeast one-hybrid (Y1H) system (Ouwerkerk and Meijer, 2001). RY-boxes of *At2S3* and *Ta1Bx7*, and mutant RY-boxes of *Ta1Bx7*^∗^ containing two repeat copies were subcloned into the vector pHis2.0 subsequently. The particular process of the experiment was described by Wang et al. (2015).

### Yeast Two-Hybrid (Y2H) Assay

The ORFs of *TaFUSCA3*, *TaFUSCA3*^∗^, *TaSPA*, *TaPBF*, and *TaGAMYB* were ligated into the pGADT7 and pGBKT7 vectors, respectively, to create the recombined plasmids (Supplementary Table S2). Of the five ORFs, *TaFUSCA3*^∗^ is a mutated version in which the seventh to the 46th amino acids were removed. For yeast mating, various combinations of different plasmids were transformed into yeast strain *Y187* by the PEG/LiAc method.

### DNA-Protein-Interaction Enzyme-Linked Immunosorbent Assay (DPI-ELISA)

The DNA-protein-interaction enzyme-linked immunosorbent assay (DPI-ELISA) assay is a versatile and effective method that was used to analyze plant TF-specific binding to DNA *in vitro*. The target protein TaFUSCA3, which was recombined with GST protein, was expressed in prokaryotic expressive *Escherichia coli* strain *BL21*. The short complementary primer sequences of biotinylation and non-biotinylation were synthesized by AuGCT DNA-SYNBiotech, Co., Ltd. (Wuhan, China) (Brand et al., 2010).

### Plant Transformation and Generation of Transgenic Plants

The intact expression cassette of *TaFUSCA3* containing the 35S promoter of cauliflower mosaic virus and the NOS terminator was amplified by reverse-transcription (RT)-PCR and cloned into the eukaryotic expression vector pBI121 to get the recombinant plasmid pBI121-*TaFUSCA3*. Subsequently, the original promoter of the *GFP* gene was replaced by the promoter of the *At2S3* gene (GenBank No.: At4g27160) that encodes the 2S albumin storage protein in *A. thaliana*. The primers are listed in Supplementary Table S1. The wild-type *A. thaliana* line *Col* and the mutant line *fus3-3* were transformed by using the *A. tumefaciens* floral dipping technique (Clough and Bent, 1998). The transgenic lines were screened on MS medium containing 50 mg/L kanamycin to generate homozygous lines. PCR and qRT-PCR analyses were performed for molecular identification and to assess transgene expression in each generation of transgenic lines.

### qRT-PCR Analysis of *TaFUSCA3*

Expression patterns of *TaFUSCA3* in different organs in wheat were analyzed by qRT-PCR (Bio-Rad, Hercules, CA, United States) using the fluorescent DNA intercalating dye SYBR Green I Master Mix. The first-strand cDNAs were synthesized from the total Poly(A)^+^ mRNA using a FastQuant RT Kit (Tiangen, Beijing, China). The gene-specific oligonucleotide primers for the gene expression analysis were designed in the 3′-UTR region of the gene excluding the B_3_ conserved domain and other highly conserved domains. To examine the efficiency and specificity, the primers were used for PCR and the products obtained were verified by sequencing as well as a melting curve analysis from 55 to 99°C. It was confirmed that a single band and peak was obtained for each primer pair. Different concentrations of various templates and primers were used to determine the optimum concentrations, and a control set of reactions was performed without template and with primer pair.

For each sample, three biological replicates were performed. The expression of *TaFUSCA3* was normalized to that of *Taβ-actin* as an internal control, and the data were analyzed based on the comparative 2^-ΔΔCT^ formula (Livak and Schmittgen, 2001).

### β-Glucuronidase (GUS) Histochemical Assay

β-Glucuronidase histochemical assay was performed with the plant tissues following the protocol of Gui et al. (2011). The samples were incubated at 37°C overnight in GUS staining buffer [100 mM sodium phosphate, 0.5 mM K_3_Fe(CN)_6_, 0.5 mM K_4_Fe(CN)_6_, 0.1% Triton X-100, 10 mM Na_2_EDTA and 1 mg/mL X-Gluc] for blue color development. After staining, the sections were rinsed with 70% ethanol for at least 1 h. Photographs were taken under fluorescence microscopy (OLYMPUS DP72, Japan).

### Western Blotting

Protein samples were electrophoresed on 12% SDS polyacrylamide gels and transferred onto nitrocellulose membranes (Millipore) for detection of the target proteins. The membranes were subsequently blocked by incubation in Tris-buffered saline plus Tween 20 (TBST: 25 mM Tris-HCl, 80 mM NaCl, 0.1% Tween 20) containing 6% fat-free milk overnight at 4°C. The membranes were incubated with the primary antibody (diluted 1:4000, 2 h), washed (10 min × 4 times), and treated with the appropriate secondary antibody for 1 h. After washing the membranes again four times with TBST, immunoreactive bands were visualized by using a chemiluminescence detection system (UVP ChemiDoc-It, Upland, CA, United States). The housekeeping β-actin protein was detected as a control (Li et al., 2012).

### Statistical Analysis

All experiments in our study were carried out in triplicate. The statistical software program ORIGIN for Windows version 8.1 was used for statistical analysis of the experimental data. Analysis of variance was used to contrast the statistically significant difference by employing the Student’s *t*-test procedure. The 5% significant differences (*P* < 0.05) and 1% remarkably significant differences (*P* < 0.01) were evaluated. Data are the means ± SD in three independent replicates (*n* = 3).

## Results

### Cloning and Sequence Analysis of the *TaFUSCA3* Gene

The cDNA of *TaFUSCA3* containing 1,071 bp was obtained from common wheat by use of an *in silico* cloning method. The *FUSCA* gene was designated *TaFUSCA3* as it had high sequence homology to *FUSCA3* (GenBank No.: AM 418838) from *H. vulgare*. The ORF of *TaFUSCA3* encoded a polypeptide of 288 amino acid residues with a predicted relative molecular mass of 32.3 kDa containing a B3 DNA binding domain. The *TaFUSCA3* gene was localized on chromosomes 3B, 3DL, and 3AL, which was also validated by the latest URGI Blast database^4^.

**FIGURE 1:**
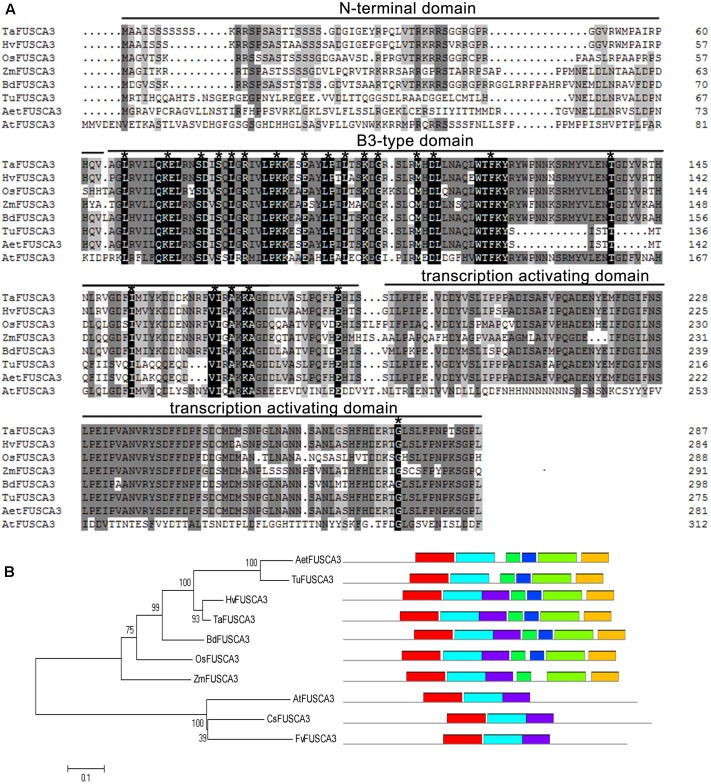
Multiple sequence alignment and phylogenetic tree analysis of TaFUSCA3. **(A)** The multiple sequence alignment was performed by use of the software DNAMAN. The extremely conserved amino acid residues in all sequences are marked with asterisks. The FUSCA3 sequences from different plant species are shown as Ta: *T. aestivum*; Hv: *H. vulgare*; Os: *O. sativa*; Zm: *Z. mays*; Bd: *B. distachyon*; Tu: *T. urartu*; Aet: *Ae. tauschii*; At: *A. thaliana*. **(B)** The phylogenetic tree was established by using the software ClustalX2.0 and MEGA5.1, which employs the minimum-evolution test and default parameters.

Multiple sequence alignment analysis indicated that TaFUSCA3 had a high sequence identity with the FUSCA3 proteins from some cereal crops (89.3% with HvFUSCA3 from *H. vulgare*, 76.3% with BdFUSCA3 from *Brachypodium distachyon*, and 68.47% with OsFUSCA3 from *Oryza sativa*), while it had lower sequence identity with FUSCA3 proteins from other monocots, such as *Triticum urartu*, *Aegilops tauschii*, and *Zea mays* (64.8, 64.2, and 61.8%, respectively) (**Figure 1A**). The protein sequences used to perform the multiple sequence alignment are shown in Supplementary Table S3. According to the Multiple EM for Motif Elicitation tool^5^, TaFUSCA3 had seven motifs, including a typical conserved B3 domain (amino acids 64–174), which could recognize and bind to the specific sequence (RY-boxes), and a transcription activating domain (TAD) containing 115 amino acid resides in the C-terminal region.

In order to further confirm the evolutionary relationships between TaFUSCA3 and FUSCA3 proteins from seven other plant species, a phylogenic tree was generated to analyze the historical evolution based on amino acid sequence alignment (**Figure 1B**). This analysis provided a further indication that FUSCA3 proteins are very highly conserved evolutionarily, and TaFUSCA3 presented the closest genetic relationship with HvFUSCA3. These results showed that *TaFUSCA3* from common wheat was a member of the FUS3 family.

### TaFUSCA3 and TaSPA Are Localized in Nucleus

**FIGURE 2:**
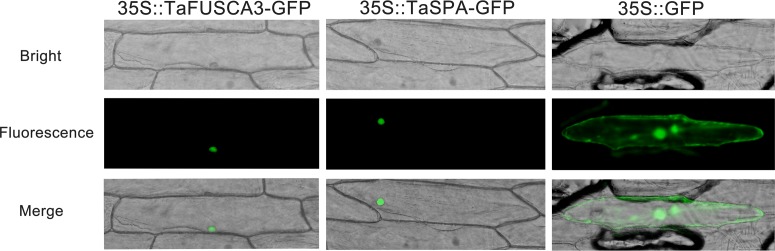
Subcellular localizations of TaFUSCA3 and TaSPA. The fusion proteins 35S::FUSCA3-GFP (pCAMBIA1303-*FUSCA3-GFP*), 35S::SPA-GFP (pCAMBIA1303-*SPA-GFP*), and 35S::GFP (pCAMBIA1303-*GFP*, control) were transiently expressed in onion epidermal cells and visualized with fluorescence microscopy 24 h after bombardment.

In order to analyze the subcellular localizations of TaFUSCA3 and TaSPA, the full-length ORFs of these proteins were fused to GFP and were transiently expressed in onion epidermal cells. As shown in **Figure 2**, both TaFUSCA3 and TaSPA were localized in the nucleus under the dark field for green fluorescence and the bright field, whereas the GFP alone was distributed throughout the cytoplasm and nucleus.

### *TaFUSCA3* are Predominantly Expressed in Stamen and Endosperm

**FIGURE 3:**
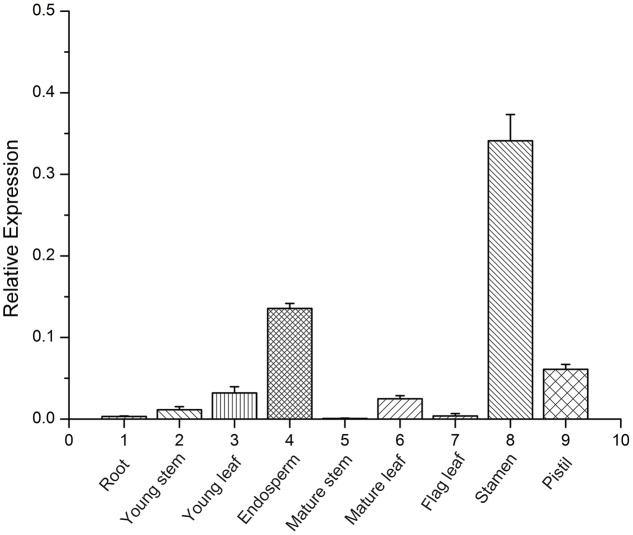
Organ-specific expression assay of *TaFUSCA3* in wheat by real-time qRT-PCR. The organs include roots, young stems, young leaves, endosperms, mature stems, mature leaves, flag leaves, stamens, and pistils. Data are means ± SD (*n* = 3) in the organ-specific expression assays. Three biological experiments showed the same results.

To define possible functions of TaFUSCA3, organ-specific expression analysis was performed by using qRT-PCR with RNA extracted from different organs including roots, young stems, young leaves, endosperms, mature stems, mature leaves, flag leaves, stamens, and pistils. The expression levels of the *TaFUSCA3* gene in different organs revealed a distinctive expression pattern, and the results showed that *TaFUSCA3* was expressed in all of the organs and had higher expression in the stamen, followed by the endosperm and pistil (**Figure 3**). This indicated that TaFUSCA3 could participate in the growth and development of wheat and in endosperm development. In addition, significantly low expression was detected in roots, stems, mature stems, and flag leaves.

**FIGURE 4:**
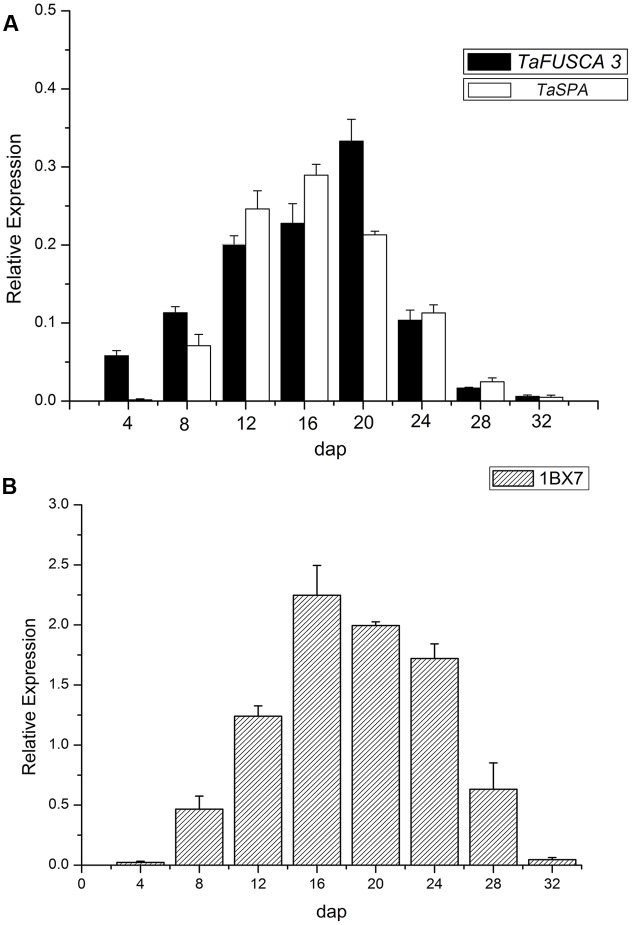
Expression analyses of the *TaFUSCA3* gene by real-time qRT-PCR. **(A)** mRNA relative expression index of *TaFUSCA3* and *TaSPA* TFs. **(B)** Expression levels of the *Ta1Bx7* gene encoding the SSP in developing endosperms from 4 to 32 DAP are shown, respectively. The *Taβ-actin* gene was used as an internal control for standardization. Error bars represent the SD for three technical replicates. Three biological experiments gave the same results. The Pearson correlation coefficient between the expression levels of *TaFUSCA3* and *TaSPA* was 0.8670 and that between *TaFUSCA3* and *Ta1Bx7* was 0.8038.

In order to further investigate the expression pattern of *TaFUSCA3* in the wheat endosperm, real-time qRT-PCR was performed with RNAs obtained from endosperms, which were isolated from the grains at different DAP (4, 8, 12, 16, 20, 24, 28, and 32). As presented in **Figure 4A**, the transcript level of *TaFUSCA3* was increased gradually from 4 DAP and peaked (sixfold) at 20 DAP, then decreased.

The expression of *TaFUSCA3* was similar to that of *TaSPA* (GenBank No.: D78609, a storage protein activator), which encodes a wheat bZIP TF of the O2-like type (Albani et al., 1997). The Pearson correlation coefficient between the expression level of *TaFUSCA3* and *TaSPA* was 0.8670, which indicated that the expression of *TaFUSCA3* was correlated positively and significantly with that of *TaSPA*. The expression pattern of *TaFUSCA3* was consistent with that of *TaSPA*, indicating that TaFUSCA3 might be a regulator of the glutenin in the developing endosperm.

The expression level of the HMW-GS gene (*Glu-1Bx*) *Ta1Bx7* was also examined by real-time qRT-PCR (**Figure 4B**). Its expression level reached a peak at 16 DAP and then reduced gradually. Wheat grain development has three growth stages: grain enlargement (10–14 days after flowering), grain fill (15–35 days after flowering), and physiological maturity. The seed increases rapidly in size as the cells that enclose the embryo sac divide and amplify during the grain enlargement stage. The grain weight increases at a constant rate as carbohydrate and protein are deposited into the grain during the grain fill stage. The SSP genes are transcribed at peak levels in the early grain fill stage in order to prepare enough mRNA templates for the next stages of carbohydrate and protein accumulation. Transcription of SSP genes is then reduced as grain development gradually comes to an end into the physiological maturity stage. The observation that *TaFUSCA3*, *TaSPA*, and *Ta1Bx7* presented almost identical expression patterns at each developmental stage suggested that *TaFUSCA3* and *TaSPA* could regulate the gene expression of the HMW-GS *Ta1Bx7* in wheat (Shewry and Halford, 2002; White and Edwards, 2008).

### TaFUSCA3 Could Specifically Recognize the RY-Box of Promoters from *Ta1Bx7* and *At2S3*

TaFUSCA3 could activate the expression of the albumin *At2S3* gene in *A. thaliana* and the glutenin *Ta1Bx7* gene in wheat by binding the *cis*-acting RY-box element (CATGCA) in the promoter specifically (**Figure 6**). To determine whether it could activate the storage protein promoter, we compared four promoter sequences of SSPs, including *At2S3*, *1Dx5*, *1Bx7*, and *1Bx13* and the sequences of them were listed in the Supplementary Table S5. The promoters of albumin *At2S3* (GenBank No.: At4g27160) in *A. thaliana* and the HMW-GS *Ta1Bx7* that have eight RY-boxes were isolated and putative control elements were screened by using PLACE^6^ (Geng et al., 2014). Yeast strains transformed with TaFUSCA3 and the RY-box from the promoters of *At2S3* and *Ta1Bx7* grew well on selective media (**Figure 6A**), which indicated that TaFUSCA3 could interact with the promoters of *At2S3* and *Ta1Bx7*.

**FIGURE 5:**
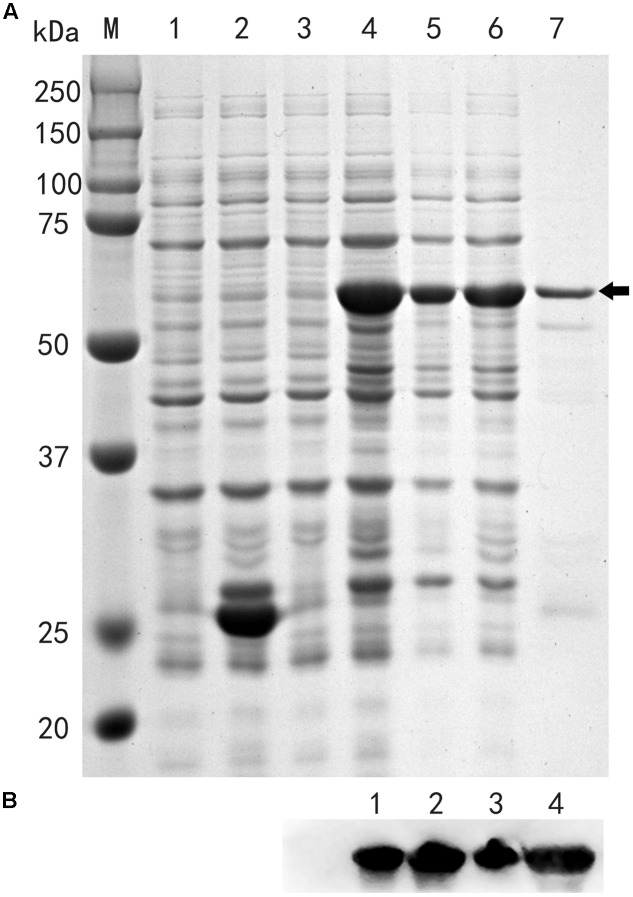
TaFUSCA3 recombined with GST protein was expressed in prokaryotic expressive *E. coli* strain BL21. **(A)** SDS-PAGE of fusion proteins isolated from the prokaryotic expression system. (1) Total proteins of strain BL21 with GST before isopropyl β-D-1-thiogalactopyranoside (IPTG) induction. (2) Total proteins of strain BL21 with GST after IPTG induction. (3) Total proteins of strain BL21 with fusion protein TaFUSCA3-GST before IPTG induction. (4) Total proteins of strain BL21 with fusion protein TaFUSCA3-GST after IPTG induction. (5) Total supernatant fluid proteins of strain BL21 with fusion protein TaFUSCA3-GST after IPTG induction. (6) Total precipitated proteins of strain BL21 with fusion protein TaFUSCA3-GST after IPTG induction. (7) Purified proteins of strain BL21 with fusion protein TaFUSCA3-GST after IPTG induction. Arrow represents the fusion protein TaFUSCA3-GST. **(B)** Western blotting verified the results of fusion protein TaFUSCA3-GST isolated from the prokaryotic expression system. (1) Total proteins of strain BL21 with fusion protein TaFUSCA3-GST after IPTG induction. (2) Total supernatant fluid proteins of strain BL21 with fusion protein TaFUSCA3-GST after IPTG induction. (3) Total precipitated proteins of strain BL21 with fusion protein TaFUSCA3-GST after IPTG induction. (4) Purified proteins of strain BL21 with fusion protein TaFUSCA3-GST after IPTG induction.

**FIGURE 6:**
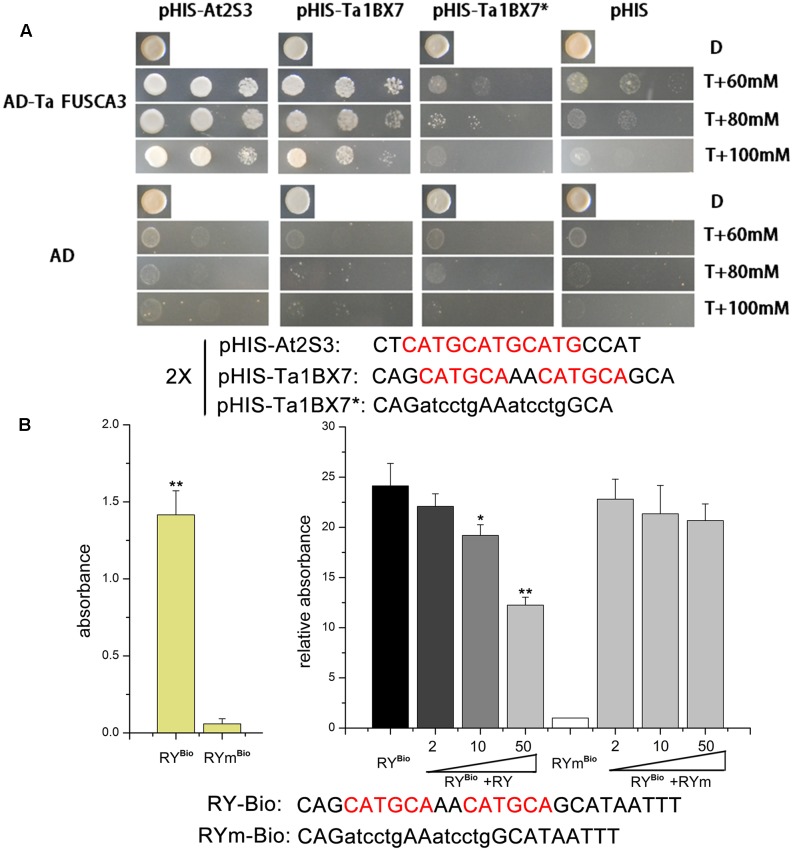
TaFUSCA3 interacts with the RY-box from the promoter of *Ta1Bx7*. **(A)** Analysis of the RY-box binding activity of TaFUSCA3 by use of the yeast one-hybrid (Y1H) assay. The effector vectors were pGAD-TaFUSCA3 and pGADT7 and reporter vectors were pHIS-At2S3, pHIS-Ta1Bx7, pHIS-Ta1Bx7^∗^, and pHIS, which was used for the Y1H system. The culture medium lacked Trp, Leu, and His with 3-amino-1,2,4-triazole (3-AT) added at a concentration of 60, 80, or 100 mM. **(B)** The specific binding property of the recombinant TF TaFUSCA3 to 5′-biotinylated dsDNA probes and competition experiment by DPI-ELISA. The RY-box probe from the promoter of *Ta1Bx7* was biotinylated at 5′ end. ELISA plates were coated with 2 pmol of double-stranded biotinylated RY Bio-probe. The specific binding of TaFUSCA3 to RY-probes was competed with non-biotinylated dsDNA in the competition experiment. Different concentrations of RY-probe or RYm-probe (0, 2, 10, 50 pmol) were added into the crude extract immediately before incubation. The biotinylated dsDNA RYmBio-probe incubated with the extract was regarded as a control. Data are means ± SD (*n* = 3) in the specific binding and competition assays. Asterisks represent statistically significant differences from the RYm-probe (0 pmol) (^∗^*P* < 0.05, ^∗∗^*P* < 0.01).

In addition, a DPI-ELISA was performed to confirm the interaction between TaFUSCA3 and the RY-box in the promoter of *Ta1Bx7*. Firstly, we successfully obtained the TaFUSCA3-GST fusion protein by use of the prokaryotic expressive assay (**Figure 5**). As presented in **Figure 6B**, TaFUSCA3 with the RY-box produced a stronger absorption signal than that with the RYm-box, which had remarkably significant difference in absorption (*P* < 0.01). The competition experiment by DPI-ELISA was performed to verify the interaction by appending different concentrations of non-biotinylated RY-probe to the binding reaction (**Figure 6B**). The increasing concentrations of non-biotinylated RY-probes competed with the binding of TaFUSCA3 to the biotinylated RY-probe successfully. The absorption signal was significantly decreased with the addition of 10 pmol non-biotinylated RY-probe (*P* < 0.05) and it was remarkably significantly reduced when the concentration of non-biotinylated RY-probe was increased up to 50 pmol (*P* < 0.01). However, the mutated RYm-probe rarely competed with the recognition of TaFUSCA3 to the biotinylated RY-probe, even at increased concentrations. Hence, specific recognition between TaFUSCA3 and the RY-box of the promoter of *Ta1Bx7* was confirmed by the Y1H and DPI-ELISA assays.

### C-Terminal Domain of TaFUSCA3 Shows Transcriptional Activity

**FIGURE 7:**
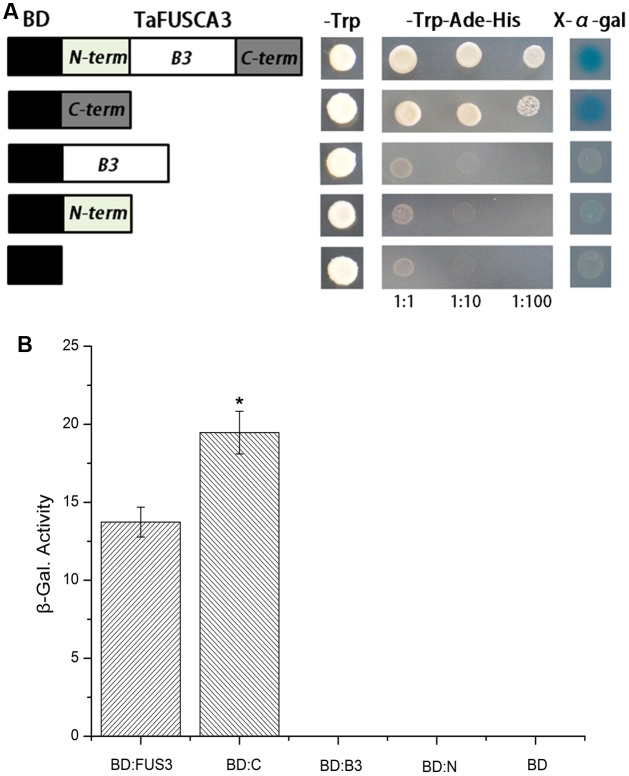
Analysis of transcriptional activity of TaFUSCA3. **(A)** The growth status of yeast strains in the Y1H assay was observed on selective medium. **(B)** The recombinant constructs including the ORF of *TaFUSCA3* and intersected fragments were transformed into the yeast strain *Y187* to analyze the transcriptional activity. *LacZ* induction was employed to evaluate activation domains within TaFUSCA3. Data are means ± SD (*n* = 3) in the Y1H system assays. Asterisks represent statistically significant differences from BD:FUS (^∗^*P* < 0.05).

Transcriptional activity of the TaFUSCA3 protein was analyzed by using the Y1H system. In order to verify which domain determines the activation capacity, yeast strain *Y187* was transformed with the recombinant plasmids containing full-length TaFUSCA3, the N-terminal region (1st to 60th amino acids), the C-terminal region (172th to 288th amino acids), and the B3 domain (61th to 171th amino acids) combined with the GAL4 binding domain, respectively. The growth status of the strain was observed on selective medium (**Figure 7A**). The yeast cells transformed with the BD-TaFUSCA3 and BD-C-terminal constructs grew well on SD medium without tryptophan, adenine, or histidine, demonstrating that TaFUSCA3 acted as a transcriptional activator. In β-galactosidase activity assay, the β-galactosidase activity of the C-terminal region was 1.4 times that of the whole protein; the other domains did not grow on the selective medium (**Figure 7B**). These results indicated that the full TaFUSCA3 and its C-terminal region had transcriptional activities.

### TaFUSCA3 Interacts with TaSPA in the Y2H System and BiFC Assay

**FIGURE 8:**
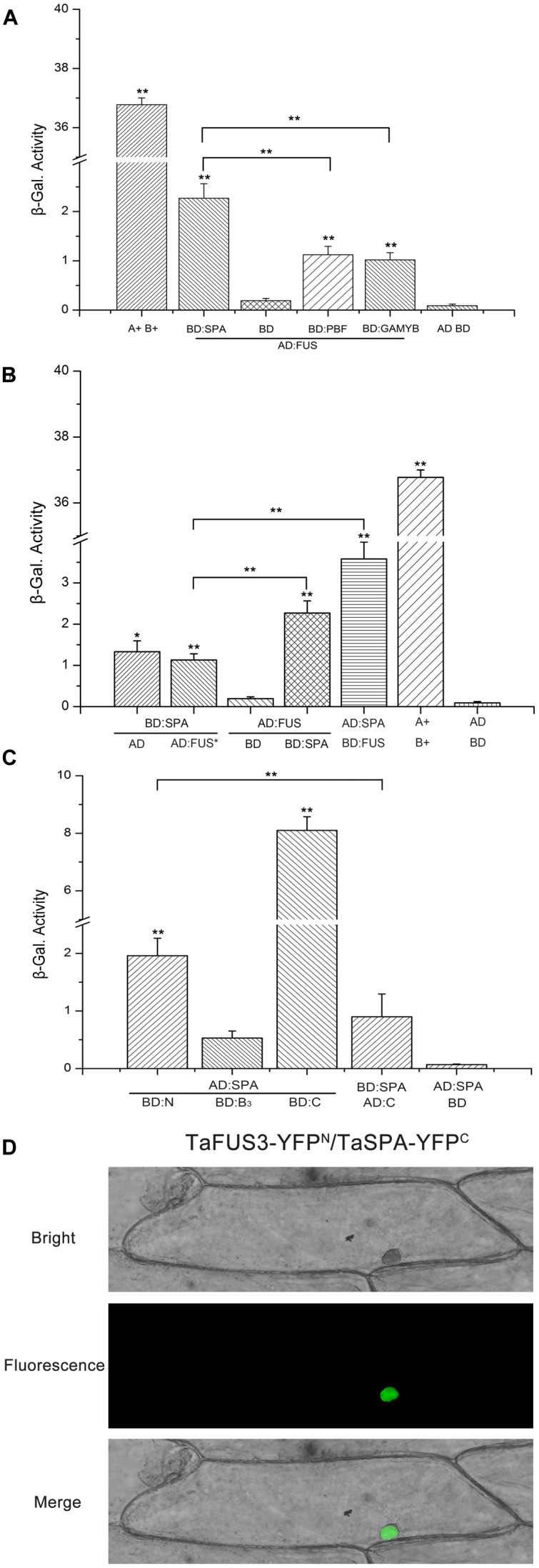
Analysis of interactions between TaFUSCA3 and TFs related to wheat storage protein expression. **(A)** Interactions of TaFUSCA3 and TaSPA, TaPBF, and TaGAMYB in the Y2H system. The indicated effector constructs were used to transform yeast strain *Y187. LacZ* induction was employed to evaluate the interaction between intersected fragments TaSPA, TaPBF, or TaGAMYB and TaFUSCA3, and β-galactosidase activity was counted from three replicates. Asterisks represent statistically significant differences from AD:FUS and BD. **(B)** Interaction of TaFUSCA3 and TaSPA in the Y2H system. The indicated effector constructs were used to transform yeast strain *Y187* containing the reporter gene *LacZ* whose promoter was Gal4. *LacZ* induction was employed to evaluate the interaction between TaFUSCA3 or TaFUSCA3^∗^ and TaSPA, and β-galactosidase activity was counted from three replicates. The positive control presented the interaction between SV40 large T-antigen and murine p53. Asterisks represent statistically significant differences from AD:FUS and BD. **(C)** Interaction of TaFUSCA3′ intersected fragments and TaSPA in the Y2H system. The indicated effector constructs were used to transform yeast strain *Y187. LacZ* induction was employed to evaluate the interaction between intersected fragments N-term, B3, or C-term and TaSPA, and β-galactosidase activity was counted from three replicates. Three biological experiments showed the same results. Asterisks represent statistically significant differences from AD:SPA and BD:B_3_. Data are means ± SD (*n* = 3) in the Y2H system assays (^∗^*P* < 0.05, ^∗∗^*P* < 0.01). **(D)** BiFC assay of TaFUSCA3. The vectors pSPYNE-*TaFUSCA3* and pSPYCE-*TaSPA* were transiently expressed in onion epidermal cells and observed by fluorescence inversion microscopy (OLYMPUS DP72, Japan) after incubation at 25°C for 24 h on MS medium in the dark.

To define the interaction of the TFs related to the regulation of wheat storage protein expression, additional cDNAs of *TaSPA* and *TaPBF* and *TaGAMYB* were cloned (Supplementary Table S4) and the Y2H assay was performed (Hu et al., 2013). For this purpose, we constructed plasmids AD-FUSCA3, AD-FUSCA3^∗^, and AD-SPA. In the meantime, we constructed effector plasmids BD-FUSCA3, BD-SPA, BD-PBF, and BD-GAMYB, in which the TFs were fused to the binding domain of the Gal4 TF. Co-transfections into the *Y187* yeast strain were performed, and the resulting transformants were tested on SD/-Lea-Trp. As shown in **Figure 8A**, yeast cells harboring AD-FUSCA3, BD, or plasmids without inserts (pGBKT7 and pGADT7) did not show β-galactosidase enzyme activity. While the β-galactosidase enzyme activity of the combination of AD-FUSCA3 and BD-SPA was remarkably significantly increased compared with that of AD-FUSCA3 with BD-PBF and AD-FUSCA3 with BD-GAMYB (*P* < 0.01). Growth was not found in the mutant *FUSCA3^∗^* (**Figure 8B**). Co-expression of *AD-SPA* with *BD-FUSCA3* resulted in very high levels of β-galactosidase, and the enzyme activity of the combination of AD-FUSCA3 and BD-SPA was roughly twofold higher than that of the group for AD-FUSCA3^∗^ and BD-SPA (*P* < 0.01), which implied that there exists an interaction between FUSCA3 and SPA.

The β-galactosidase activity assay further confirmed interactions between SPA and the N-terminal domain of FUSCA3 in yeast (**Figure 8C**). TaSPA was used as prey to interact with the N-terminal domain, B3 domain, and C-terminal domain from TaFUSCA3. For the N-terminal domain, the interaction with SPA was roughly fourfold greater than that of the B3 domain (*P* < 0.01). Because the C-terminal domain had a transcriptional activation activity, it showed a higher β-galactosidase activity when used as bait. But it presented a lower enzyme activity when used as prey.

To further confirm the interaction between TaFUSCA3 and TaSPA, a BiFC assay was performed in epidermal cells of onions. The C-terminal fragment of yellow fluorescent protein (YFP) fused with TaFUSCA3 was able to interact with the N-terminal fragment of YFP fused with TaSPA in the nucleus (**Figure 8D**). The fluorescence signal was found only in the nucleus. These results indicated that TaFUSCA3 interacts with TaSPA and the complex is colocalized in the nucleus.

### TaFUSCA3 Activates Transcription of the *At2S3* and *Ta1Bx7* Genes

**FIGURE 9:**
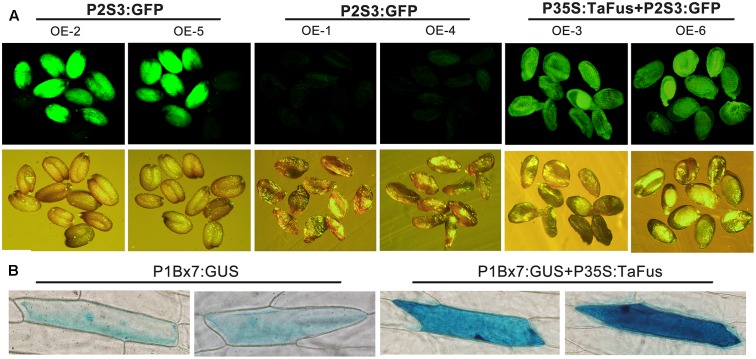
Complementation of TaFUSCA3 in the *A. thaliana FUSCA3* mutant line *fus3-3* and transcriptional regulation of the promoter of Ta1Bx7 in a transient expression system. **(A)** The *GFP* gene under the control of the *At2S3* promoter and the *TaFUSCA3* gene driven by the *CaMV 35S* promoter were transformed into wild-type and *fus3-3* lines of *A. thaliana*, respectively. The genetic background of OE-2 and OE-5 was wild-type; that of OE-1, OE-4, OE-3, and OE-6 was the *FUSCA3* mutant line *fus3-3.* The seeds of transgenic lines were observed under a fluorescence microscope to evaluate the expression level of the *At2S3* promoter. **(B)** The *GUS* gene under the control of the *Ta1Bx7* promoter and the *TaFUSCA3* gene driven by the *CaMV 35S* promoter, respectively, were transformed into onion epidermal cells in a transient expression system.

To verify the inference about conservation of function between TaFUSCA3 and AtFUSCA3, the *A. thaliana* wild-type *Col* and mutant type *fus3-3* were transformed with *TaFUSCA3* (Keith et al., 1994). The expression cassette including CDS (coding sequence) of *TaFUSCA3*, 35S promoter, and NOS terminator was inserted into pBI121 in which the *GFP* gene was driven by the promoter of *At2S3*. The results of PCR and qRT-PCR for the molecular identification and detection of transgene expression in the transgenic lines are shown in **Supplementary Figures S1**, **S2**. The expression level of albumin 2S had been confirmed to be strongly decreased in the grains of the *FUSCA3* mutant line (Luerssen et al., 1998). The mature seeds of wild-type *Col* and mutant type *fus3-3* transformed respectively with the *GFP* gene under the control of the *At2S3* promoter and TaFUSCA3 were observed by fluorescence inversion microscopy (OLYMPUS DP72, Japan). As shown in **Figure 9A**, fluorescence intensity of GFP, which was weakened intensively in the *fus3-3* lines, was recovered to nearly the same level as that of the wild-type in the grains expressing *TaFUSCA3* constitutively.

The transient expression of *TaFUSCA3* in onion epidermal cells was used to confirm the transcription activation of the *Ta1Bx7* gene promoter (**Figure 9B**). The GUS staining of the onion epidermal cells revealed that expression level of *GUS* driven by the *Ta1Bx7* promoter was obviously increased following co-transformation with the *TaFUSCA3* gene. It could be inferred that TaFUSCA3 activated the transcription of the *Ta1Bx7* promoter, which led to the increase of expression of *GUS*.

## Discussion

FUSCA3 is a master regulator for seed growth, including lipid accumulation, establishment of developmental timing, and identity development of lateral organs, as well as playing a role in the formation of SSPs and seed maturation (Reidt et al., 2001; Gazzarrini et al., 2004; Tsai and Gazzarrini, 2012). Both the quantity and quality of SSPs can affect flour quality in wheat. Higher dough strength usually predicts better-quality bread in the process of wheat breeding (Cooper et al., 2016). Although the functional importance of FUSCA3 has been studied in *A. thaliana* and other model plants, the structural and functional characteristics of FUSCA3 in wheat, especially in the transcriptional regulation of SSPs, have not yet been explored.

We cloned a FUS3CA-like transcriptional factor gene designated *TaFUS3CA3* from *T. aestivum* in this study. Our results show that *TaFUS3CA3* is the putative paralogous gene of *HvFUSCA3* from barely. Both genes not only have high homology phylogenetically, but also can exert analogous functions. *TaFUSCA3* was expressed in different tissues, but it had highest expression in the endosperm. We compared three promoter sequences of wheat storage proteins, including *1Dx5*, *1Bx7*, and *1Bx13* and the sequences of them were listed in the Supplementary Table S5. It is confirmed that TaFUSCA3 can activate the promotion of the HMW-GS gene *Ta1Bx7*. Our results showed that expression patterns of *TaFUSCA3* and the storage protein gene *Ta1Bx7* were similar in the wheat endosperm. The Pearson correlation coefficient between the expression levels of *TaFUSCA3* and *Ta1Bx7* was 0.8038, which indicated that the expression of *TaFUSCA3* was correlated significantly with that of *Ta1Bx7*. The SSPs of cereals are accumulated in the endosperms, whereas those of *A. thaliana* are accumulated in the cotyledons (Santos-Mendoza et al., 2008). Therefore, the distinct functions of FUSCA3 in the two different species should be explored further.

Expressional regulation of target genes is usually manifested by the synergistic effects of various TFs. The expression level of a gene could be attributed to the regulating effects of multiple TFs by recognizing a specific promoter cooperatively. Many significant interactions (such as O2-PBF, BLZ2-BLZ1, SAD-GAMYB, AKIN10-FUSCA3, and GAMYB-VP1) have been identified in the regulation of target genes in some species (Nakamura et al., 2001; Diaz et al., 2005; Tsai and Gazzarrini, 2012; Abraham et al., 2016). Further studies are still necessary to explore the interactions between TaFUSCA3 and other relevant TFs in wheat.

In order to define the TFs that interacts with TaFUSCA3, the TFs related to the regulation of wheat storage protein expression including TaSPA, TaPBF, and TaGAMYB were screened (**Figure 8A**). Our data revealed that TaFUSCA3 can interact with TaSPA (**Figure 8B**), and the protein–protein interactions between TaFUSCA3 and TaSPA increased the β-galactosidase activity remarkably significantly in the Y2H assay (*P* < 0.01). Furthermore, the mutated type TaFUSCA3^∗^ was not able to interact with TaSPA (**Figure 8B**), and TaFUSCA3 could interact with TaSPA through the N-terminal domain (**Figure 8C**). The BiFC assay further provided evidence for the interaction between TaFUSCA3 and TaSPA (**Figure 8D**). The expression level of TaFUSCA3 was consistent with that of TaSPA, indicating that TaFUSCA3 was a regulator of the glutenin in the developing endosperm (**Figure 4**). TaFUSCA3 could activate the expression of the albumin *At2S3* gene in *A. thaliana* and the glutenin *Ta1Bx7* gene in wheat by specifically binding the *cis*-acting RY-box element (CATGCA) from their promoters in the Y1H and DPI-ELISA assays (**Figure 6**). The interactions between TaFUSCA3 and TaSPA TFs shown in our study propose the further exploration of the regulatory mechanism of SSPs in wheat. It is apparent that the regulation of expression of stored energy substances in wheat can be attributed to complicated interactions of TFs.

It was reported that AtFUSCA3 can activate transcription by recognizing the sequence 5′-CATGCA-3′ (RY-box) of SSP gene promoters (Wang and Perry, 2013). Furthermore, other reports suggest that FUSCA3 indirectly modulates the expression of seed storage reserve genes through suppressing formation of TTG1, which is related to epidermal morphogenesis (Tsuchiya et al., 2004; Wang et al., 2014; Chen et al., 2015). Here, we showed that TaFUSCA3 could specifically recognize and combine with the RY-box (5′-CATGCA-3′), which comes from the promoters of the *Ta1Bx7* and *At2S3* genes, through the B3 domain and activate expression of these genes through the C domain (**Figures 6**, **7**). We also carried out the reciprocal assay in which the RY-box was mutated, which prevented TaFUSCA3 from recognizing the promoter of the *Ta1Bx7* gene and destroyed the TaFUSCA3-mediated activity of transcriptional activation (**Figure 6**). Our results indicated that transcriptional activation of *Ta1Bx7* and *At2S3* resulted from the immediate recognition and binding of the RY motif of promoters by TaFUSCA3.

The objective of our research was to clone the *FUSCA3* gene in wheat and to identify its function in the control of expression of wheat SSP. A wheat cDNA was cloned, which was the homologous gene to *HvFUSCA3*. When the ORF of *TaFUSCA3* was transformed into *A. thaliana* line *fus3-3* with *AtFUSCSA3* mutated, we found that characteristics were recovered in transgenic plants, such as activated expression of the promoter from the SSP gene *At2S3* (**Figure 9A**). TaFUSCA3 could restore the mutant feature of *fus3-3*, which indicates that it possibly takes part in the same regulatory pathways of the SSPs by acting in the same role as AtFUSCA3. It might perform these functions by combining with the other regulatory factors and forming a transcription initiation complex.

The composition and quantity of SSPs can dramatically affect dough quality in wheat, and the regulation of expression is important to control in the formation of SSPs (Wieser, 2000). TaFUSCA3 was able to activate transcription of At2S3, which suggested to us to consider whether the *FUSCA3* gene from wheat was also related to the transcriptional regulation of SSP in wheat. We explored the inference by analyzing the promoters of storage proteins in wheat that include conserved base sequences (RY-box) for TaFUSCA3 (*1Dx5*, *1Bx7*, and *1Bx13*). We found that TaFUSCA3 could *trans*-activate the promoter of the *Ta1Bx7* gene encoding the HMW-GS by recognizing a necessary RY motif component in the storage protein gene (**Figures 6**, **9**), which suggested that we might be able to improve the SSP content by overexpressing *TaFUSCA3* and *TaSPA* in grains. Regulation of the wheat storage protein gene by TaFUSCA3 and its compensatory effect in the mutant line *fus3-3* in *A. thaliana* reflect the conservation of the evolution of this gene between the dicotyledonous and monocotyledonous SSP. The conservation not only is reflected in the structure of proteins but also in the *cis*-action and *trans*-action regulation (Lara et al., 2003).

## Conclusion

*TaFUSCA3* was identified and cloned in wheat. The structure and functional characteristics of wheat B3-type TF TaFUSCA3 were explored in the aspects of sequence alignment and expression pattern, as well as the protein interactions and transcriptional regulation in wheat SSPs. TaFUSCA3 can specifically recognize and combine with the RY-box (5′-CATGCA-3′) of the promoters from the *Ta1Bx7* and *At2S3* genes through the B3 domain and activate expression of these genes through the C domain. Moreover, it was shown that TaFUSCA3 interacted with the seed-specific bZIP protein TaSPA through the N domain. TaFUSCA3 can complement loss-of-function in the *A. thaliana FUSCA3* mutant line *fus3-3* by regulating SSP gene expression in seeds. The structure and functional characteristics of wheat B3-type TF TaFUSCA3 were explored in the aspects of sequence alignment and expression pattern, as well as the protein interactions and transcriptional regulation in wheat SSPs. Although the function of *TaFUSCA3* was not verified in transgenic wheat, the findings of this study shed light on the regulatory mechanism and increased abundance of SSP in wheat.

## Author Contributions

GH, GY, and FS designed the experiments and wrote the paper. FS and XL performed all experiments and analyzed the data. TY and LJ helped to construct the relevant vectors. QW and JL helped to conduct the experiments. YW participated in partial experiments.

## Conflict of Interest Statement

The authors declare that the research was conducted in the absence of any commercial or financial relationships that could be construed as a potential conflict of interest.

## Footnotes

1 http://plants.ensembl.org/Triticum_aestivum/Info/Index

2 https://www.ncbi.nlm.nih.gov/orffinder

3 https://www.ncbi.nlm.nih.gov

4 https://urgi.versailles.inra.fr/blast/blast.php

5 http://meme-suite.org

6 http://www.dna.affrc.go.jp/PLACE/signalup.html

## Supplementary Material

**FIGURE S1 |** The molecular identification by PCR for transgenic lines in *A. thaliana*. **(A)** The transgenic lines with *GFP* gene under the control of the *At2S3* promoter and genetic background was wild type. WT: wild type. The primer used was GFP1 listed in the Supplementary Table S1. **(B)** The transgenic lines with *GFP* gene under the control of the *At2S3* promoter and genetic background was *AtFUSCA3* mutant line *fus3-3*. MT: non-transgenic mutant type. The primer used was GFP1 listed in the Supplementary Table S1. **(C)** Transgenic lines with the *GFP* gene under the control of the *At2S3* promoter and the *TaFUSCA3* gene driven by the *CaMV 35S* promoter; the genetic background was the *AtFUSCA3* mutant line *fus3-3*. MT: non-transgenic mutant type. The primer used was GFP1 listed in the Supplementary Table S1. **(D)** Transgenic lines with the *GFP* gene under the control of the *At2S3* promoter and the *TaFUSCA3* gene driven by the *CaMV 35S* promoter; the genetic background was the *AtFUSCA3* mutant line *fus3-3*. MT: non-transgenic mutant type. The primer used was JDfusca3 listed in Supplementary Table S1.

**FIGURE S2 |** The expression analyses of GFP and TaFUSCA3 by qRT-PCR in transgenic *A. thaliana*. The 14 DAP seeds were collected for the cDNA of qRT-PCR. **(A)** Relative expression levels of GFP in the transgenic lines with *GFP* gene; the genetic background was wild type. WT: wild type. **(B)** Relative expression levels of GFP in the transgenic lines with *GFP* gene; the genetic background were *AtFUSCA3* mutant line *fus3-3*. MT: non-transgenic mutant type. **(C)** Relative expression levels of GFP in the transgenic lines with GFP and TaFUSCA3; the genetic background were *AtFUSCA3* mutant line *fus3-3*. MT: non-transgenic mutant type. **(D)** Relative expression levels of TaFUSCA3 in the transgenic lines with GFP and TaFUSCA3; the genetic background was the *AtFUSCA3* mutant line *fus3-3*. MT: non-transgenic mutant type. Data are means ± SD (*n* = 3). Three independent experiments were performed.

## Abbreviations

BiFC: bimolecular fluorescence complementation
DAP: days after pollination
DPI-ELISA: DNA-protein-interaction enzyme-linked immunosorbent assay
GFP: green fluorescent protein
GST: glutathione *S*-transferase
GUS: β-glucuronidase
HMW-GS: high molecular weight glutenin subunit
O2: opaque2
ORF: open reading frame
qRT: quantitative reverse-transcription
SSPs: seed storage proteins
TFs: transcription factors
Y1H: yeast one-hybrid
Y2H: yeast two-hybrid
