# Supplementary figures and images for: Functional Characterization of TaFUSCA3, a B3-Superfamily Transcription Factor Gene in the Wheat
Source: Front Plant Sci. 2017 Jun 28;8:1133. doi: 10.3389/fpls.2017.01133 (PMC5487486; doi:10.3389/fpls.2017.01133)

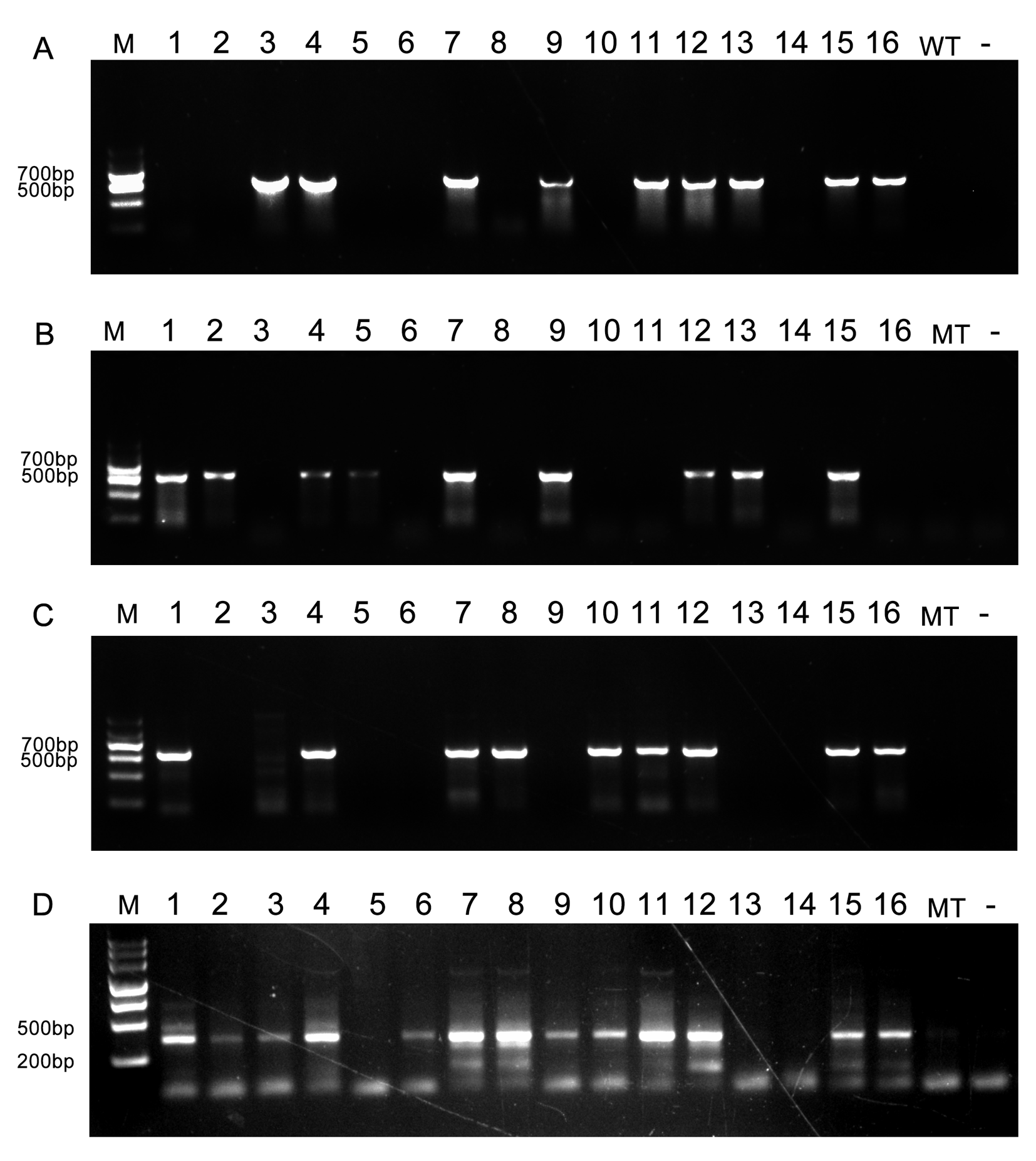

Supplement: Supplementary file 1 [file Image_1.TIF]

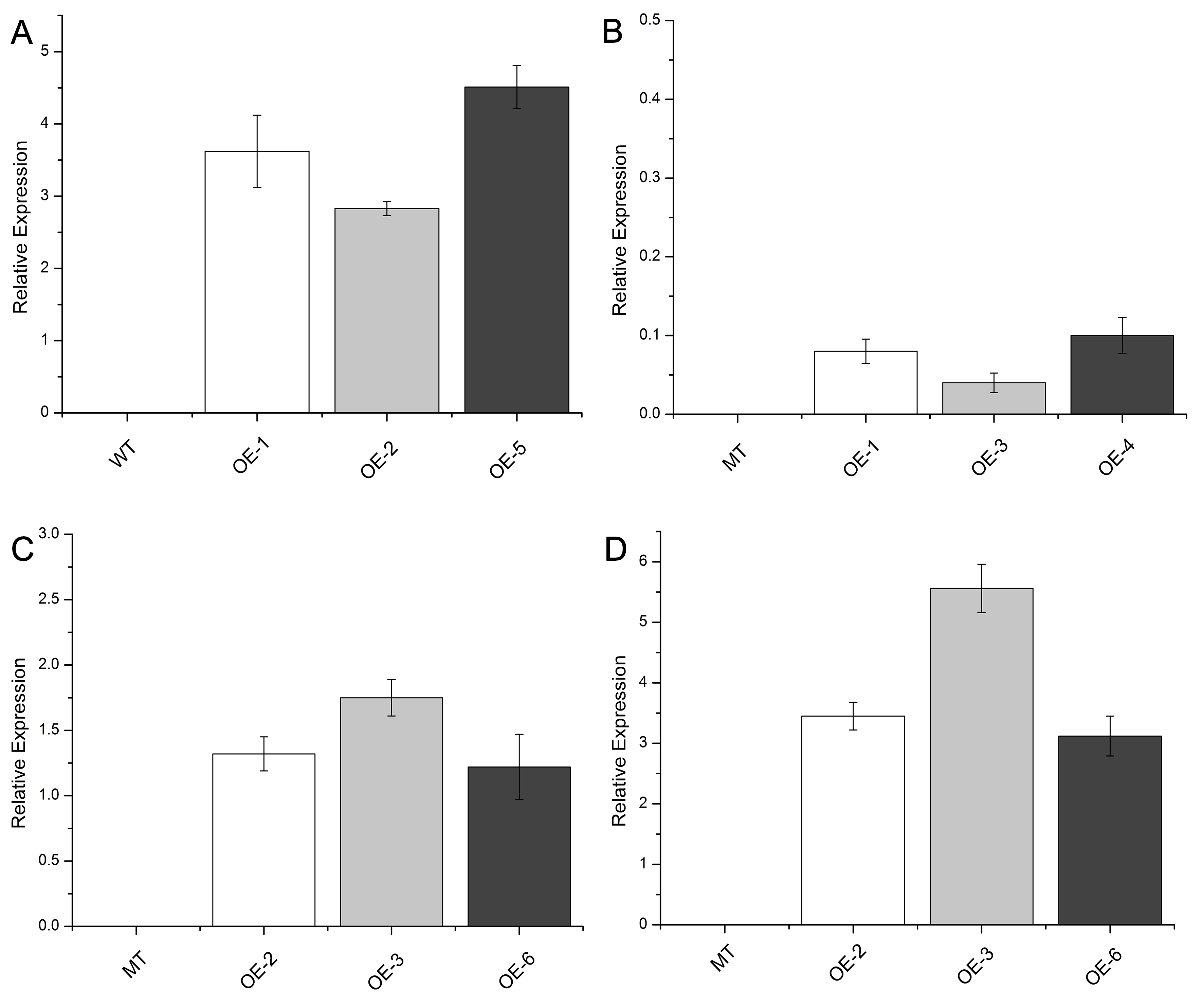

Supplement: Supplementary file 2 [file Image_2.TIF]
