# Supplementary material for: Functional Characterization of TaFUSCA3, a B3-Superfamily Transcription Factor Gene in the Wheat
Source: Front Plant Sci. 2017 Jun 28;8:1133. doi: 10.3389/fpls.2017.01133 (PMC5487486; doi:10.3389/fpls.2017.01133)
Supplement: Supplementary file 3 [file Table_1.DOCX]

**Supplementary Table S1** Forward and reverse primers used in gene clone, expression analyses and vector constructions

| Gene Names | Primer Name | | Forward primers | Reverse primers | **Assays** |
| --- | --- | --- | --- | --- | --- |
| *TaFUSCA3* | P1 | acccatctcgcccataaa | | caaggctggtgactctgaac | clone of gene |
| *TaSPA* | P2 | gcccaattttgttctaatcaatca | | gccgagaaaccactataaataccc | clone of gene |
| *TaPBF* | P3 | cacctatactccatactac | | ttacatcagggaggtgctgttga | clone of gene |
| *TaGAMYB* | P4 | atgtaccgggtgaagagcga | | tcatttgaattcctccga | clone of gene |
| *TaFUSCA3* | P5 | cggtagccaatgtgaggt | | atggtaggagccagcagt | genes expression analyses |
| *TaSPA* | P6 | tccaaggctcagaaaacca | | ctcgccgaatccctgtta |  |
| *GFP*2 | P7 | tccacacaatctgccctttc | | tgcgggactctaatcataaaaa |  |
| *TaActin* | P8 | tctattttggcctctcttagcac | | tttcctgtaccccttattcctc |  |
| *AtActin* | P9 | ggtaacattgtgctcagtggtgg | | aacgaccttaatcttcatgctgc |  |
| *TaFUSCA3* | P10 | CCGGAATTCatggccgccatcagcagcagca | | CGCGGATCCcatcagaggcccagacgttgggt | Y2H assays |
| *TaSPA* | P11 | CCGGAATTCatggagcccgtgttcttctca | | CGCGGATCCcaccatgttgactatttcag |  |
| *TaPBF* | P12 | CCGGAATTCatggaggaagtgtttccgtcaaa | | CGCGGATCCttacatcagggaggtgctgttga |  |
| *TaGAMYB* | P13 | CATGCCATGGATatgtaccgggtgaagagcga | | ACGCGTCGACtcatttgaattcctccga |  |
| *TaFUSCA3^*^* | P14 | CCGGAATTCatggccgccatcagcagcgggccccgcggcggcgtc | | CGCGGATCCcatcagaggcccagacgttgggt |  |
| *TaFUSCA3* | P15 | CGCGGATCCatggccgccatcagcagcagca | | CGGGGTACCcatcagaggcccagacgttgggt | BiFC |
| *TaSPA* | P16 | CGCGGATCCatggagcccgtgttcttctca | | CGGGGTACCcatcaccatgttgactattt |  |
| *TaFUSCA3-BD* | P17 | CCGGAATTCatggccgccatcagcagcagca | | CGCGGATCCcatcagaggcccagacgttgggt | transcriptional activity analyses |
| *N-BD* | P18 | CCGGAATTCatggccgccatcagcagca | | CGCGGATCCagccacttgatgtggtcgg |  |
| *B_3_-BD* | P19 | CCGGAATTCgggttgcgagttattctg | | CGCGGATCCatcatctcccgccttctt |  |
| *C-BD* | P20 | CCGGAATTCctagttgcttctttgccac | | CGCGGATCCtcacatcagaggcccaga |  |
| *TaFUSCA3* | P21 | CCGGAATTCatggccgccatcagcagcagca | | CCGCTCGAGctacatcagaggcccagacg | expression of fusion protein |
| *TaFUSCA3* | P22 | CATGCCatggccgccatcagcagcagca | | CGGACTAGTcatcagaggcccagacgttgggt | Subcellular localization |
| *TaSPA* | P23 | CATGCCatggagcccgtgttcttctca | | CGGACTAGTcaccatgttgactatttcag |  |
| *TaFUSCA3* | P24 | AAGCTTtgagacttttcaacaaag | | AAGCTTgatctagtaacatagatga | transformation in Arabidopsis |
| *At2S3* | P25 | AAGCTTaaaccaaattaacatagg | | GGATCCgttttgctatttgtgtatg |  |
| *JDfusca3* | P26 | attgcccagctatctgtcactt | | ggtactcgccgattccgt | positive strains identification |
| *GFP*1 | P27 | ctgtcagtggagagggtgaagg | | cgaaagggcagattgtgtgg |  |
| *TaFUSCA3*  *1Bx7* | P28  P29 | CGCGGATCCatggccgccatcagcagcagca  CTGCAGgttgacgggtcgtttcgcgc | | GGTACCtcacatcagaggcccagac  AGATCTaccatgagtcacttgacagtc | transient expression |
