# Supplementary material for: Functional Characterization of TaFUSCA3, a B3-Superfamily Transcription Factor Gene in the Wheat
Source: Front Plant Sci. 2017 Jun 28;8:1133. doi: 10.3389/fpls.2017.01133 (PMC5487486; doi:10.3389/fpls.2017.01133)
Supplement: Supplementary file 4 [file Table_2.DOCX]

**Supplementary Table S2** The table for vectors used in this study.

| Gene Names | Vector Names | Assays |
| --- | --- | --- |
| *TaFUSCA3* | pMD18-T-*TaFUSCA3* | clone of gene |
| *TaSPA* | pMD18-T-*TaSPA* | clone of gene |
| *TaPBF* | pMD18-T-*TaPBF* | clone of gene |
| *TaGAMYB* | pMD18-T*-TaGAMYB* | clone of gene |
| *TaFUSCA3* | pGADT7-*TaFUSCA3*, pGBKT7-*TaFUSCA3* | Y2H assays |
| *TaSPA* | pGADT7-*TaSPA*, pGBKT7-*TaSPA* |  |
| *TaPBF* | pGBKT7-*TaPBF* |  |
| *TaGAMYB* | pGBKT7-*TaGAMYB* |  |
| *TaFUSCA3^*^* | pGADT7-*TaFUSCA3^*^* |  |
| *TaFUSCA3* | pSPYNE-*TaFUSCA3* | BiFC |
| *TaSPA* | pSPYCE-*TaSPA* |  |
| *TaFUSCA3* | pGBKT7-*TaFUSCA3* | transcriptional activity analyses |
| *TaFUSCA3-N* | pGBKT7-*TaFUSCA3-N* |  |
| *TaFUSCA3-B_3_* | pGBKT7-*TaFUSCA3-B_3_* |  |
| *TaFUSCA3**-C* | pGBKT7-*TaFUSCA3-C* |  |
| *TaFUSCA3*  *1Bx7-promoter*  *At2S3-promoter* | pGADT7-*TaFUSCA3* | binding activity analysis |
|  | pHIS-1Bx7-*2×RY* |  |
|  | pHIS-2S3-*2×RY* |  |
|  | pHIS-1Bx7-*2×RY^*^* |  |
| *TaFUSCA3* | pCAMBIA1303-*TaFUSCA3*-*GFP* | Subcellular localization |
| *TaSPA* | pCAMBIA1303-*TaSPA-GFP* |  |
| *TaFUSCA3*  *At2S3-promoter* | pBI121-*TaFUSCA3* | transformation in Arabidopsis |
|  | pBI121-2S3-*GFP* |  |
|  | pBI121-*TaFUSCA3*-2S3-*GFP* |  |
| *TaFUSCA3* | pSN1301-1Bx7*-GUS* | transient expression |
| *1Bx7-promoter* | pSN1301-*TaFUSCA3-*1Bx7*-GUS* |  |
| *TaFUSCA3* | pGEX-4T-1-*TaFUSCA3* | expression of fusion protein |
