# Supplementary material for: Functional Characterization of TaFUSCA3, a B3-Superfamily Transcription Factor Gene in the Wheat
Source: Front Plant Sci. 2017 Jun 28;8:1133. doi: 10.3389/fpls.2017.01133 (PMC5487486; doi:10.3389/fpls.2017.01133)
Supplement: Supplementary file 5 [file Table_3.DOCX]

**Supplementary Table S3** The protein sequences of FUSCAS3s for multiple sequence alignment.

>TaFUSCA3

MAAISSSSSSSKRRSPSASTTSSSSGDGIGEYRPQLVTRKRRSGGRGPRGGVRWMPAIRPHQVAGLRVILQKELRNSDISQLGRIVLPKKESEAYLPILTSKDGRSLRMHDLLNAQLWTFKYRYWPNNKSRMYVLENTGDYVRTHNLRVGDFIMIYKDDDKNRFVIRAKKAGDDLVASLPQFHEHISSILPIPEVDDYVSLIPPPADISAFVPQADENYEMFDGIFNSLPEIPVANVRYSDFFDPFSDCMDMSNPGLNANNSANLGSHFHDERTGLSLFPNPTSGPLM

>HvFUSCA3(CAL91173)

MAAISSSKRRSPSASTAASSSSADGIGEPGPQLVTRKRRSVRRGPRGGVRWMPAIRPHQVPGLRVILQKELRNSDISQLGRIVLPKKESEAYLPTLASKDGRSLRMHDLLNAQEWTFKYRYWPNNNSRMYVLENTGDYVRTHNLRVGDFIMVYKDDDNNRFVIRAKKAGDDLVAAMPQFHEHTSGILPIPEVDDYVSLIPSPADISAFIPQADENYEIFDGIFNSLPEIPVANVRYSDFFDPFSDCMDASNPSLNGNNSANLASHFHDERTGLSLFPNPKSGPLM

>OsFUSCA3(Os01g5161)

MAGVTSKRRSSSASTSSSSGDGAAVSDRPRGVTRKRRSGGRCPRPAASLRPAAPRPSSHHTAGLRVILQKELRYSDVSQLGRIVLPKKEAEAYLPILTSKDGKKSLCMHDLQNAQLWTFKYRYWPNNKSRMYVLENTGDYVRTHDLQLGDSIVIYKDDENNRFVIGAKKAGDQQAATVPQVDEHISTLFPIFPIAQVDDYLSPMAPQVDISAFVPHADENHEIFDGILNSLPEIPVANVRYSDFFDPFDDGMDMANTLNANANQSASLHVTDDKSGHSLIPNPKSGPHM

>ZmFUSCA3(AFW83736)

MAGITKRRTSPASTSSSSGDVLPQRVTRKRRSARRGPRSTARRPSAPPPMNELDLNTAALDPDHYATGLRVLLQKELRNSDVSQLGRIVLPKKEAESYLPILMAKDGKSLCMHDLLNSQLWTFKYRYWFNNKSRMYVLENTGDYVKAHDLQQGDFIVIYKDDENNRFVIGAKKAGDEQTATVPQVHEHMHISAALPAPQAFHDYAGPVAA

EAGMLAIVPQGDEIFDGILNSLPEIPVANVRYSDFFDPFGDSMDMANPLSSSNNPSVNLATHFHDERIGSCSFPYPKSGPQM

>BdFUSCA3(XP014754315)

MDGVSSKRRSPSASSTSTSSGDVTSAARTQRVTRKRRSGGRGPRRGGLRRPPAHRPVNEMDLNRAVFDPDHQVLAGLHVILQKELRNSDISQLGRIILPKKEAEAYLPILTSKDGKSLCMHDLLNAQLWTFKYRYWPNNKSRMYVLENTGDYVRAHNLQVGDFIMIYKDDNNNRFVIRAKKAGDDLAATVPQIDEHISVMLPKPEVDDYMSLISPQADISAFMPQADENYEIFDGILNSLPEIPAANVRYSDFFNPFDDSMDMSNPGLNANNSVNLMTHFHDDKAGLSLFPNPKSGPLI

>TuFUSCA3(EMS68624)

MRTIHQQAHTSNSGERGEGPNYLREGEEVVDLTTQGGSDLRAADGGELCMTLHVNELDLNRVALDPNHQVAGLRVILQKELRNSDISQLGRIVLPKKESEAYLPILTSKDGRSLRMHDLLNAQLWTFKYSISTTMTQFIISVQILAQQQEQDVIRAKKAGDDLVASLPQFHEHISSILPIPEVGDYVSLIPPPADISAFAPQADENYEMFDGIFNSLPEIPVANVRYSDFFDPFSDCMDMSNPGLNANNSANLASHFHDERTGLSLFPNPKSGPLM

>AetFUSCA3(EMT15116)

MGRAVPCRAGVLLNSTIRFHPPSVRKLGKLSVLFLSSLRGEKLCERSIIYITTMMDRTGVNELDLNRVSLDPNHQVAGLRVILQKELRNSDISQLGRIVLPKKESEAYLPILTSKDGRSLRMHDLLNAQLWTFKYSISTTMTQFIISVQILAKQQEQDVIRAKKAGDDLVASLPQFHEHISSILPIPEVDDYVSLIPPPADISAFVPQADENYEMFDGIFNSLPEIPVANVRYSDFFDPFSDCMDMSNPGLNANNSANLASHFHDERTGLSLFPNPKSGPLM

>AtFUSCA3(AAC35247)

MVDENVETKASTLVASVDHGFGSGSGHDHHGLSASVPLLGVNWKKRRMPRQRRSSSSFNLLSFPPPMPPISHVPTPLPARKIDPRKLRFLFQKELKNSDVSSLRRMILPKKAAEAHLPALECKEGIPIRMEDLDGFHVWTFKYRYWPNNNSRMYVLENTGDFVNAHGLQLGDFIMVYQDLYSNNYVIQARKASEEEEVDVINLEEDDVYTNLTRIENTVVNDLLLQDFNHHNNNNNNNSNSNSNKCSYYYPVIDDVTTNTESFVYDTTALTSNDTPLDFLGGHTTTTNNYYSKFGTFDGLGSVENISLDDFY
