# Supplementary material for: Functional Characterization of TaFUSCA3, a B3-Superfamily Transcription Factor Gene in the Wheat
Source: Front Plant Sci. 2017 Jun 28;8:1133. doi: 10.3389/fpls.2017.01133 (PMC5487486; doi:10.3389/fpls.2017.01133)
Supplement: Supplementary file 6 [file Table_4.DOCX]

**Supplementary Table S4** The ORF sequences of wheat translation factor *Ta FUSCA3*,*TaSPA*, *TaPBF* and *TaGAMYB*.

>*TaFUSCA3*

ATGGCCGCCATCAGCAGCAGCAGCAGCAGCAGCAAGCGCCGCTCCCCCTCCGCATCCACCACCTCCTCCTCCTCCGGCGACGGAATCGGCGAGTACCGGCCGCAGCTGGTCACCCGGAAGCGGAGGTCCGGCGGCCGCGGGCCCCGCGGCGGCGTCCGGTGGATGCCGGCGATCCGACCACATCAAGTGGCTGGGTTGCGAGTTATTCTGCAGAAGGAGCTCCGTAATAGTGACATAAGCCAGCTTGGGAGAATTGTTCTCCCGAAGAAAGAATCGGAGGCTTACCTCCCAATTCTGACATCAAAGGATGGCAGAAGTCTACGCATGCATGATTTGCTAAATGCGCAACTGTGGACATTCAAGTACAGATACTGGCCGAACAACAAGAGCAGGATGTATGTACTTGAGAATACTGGGGATTATGTCCGAACCCATAACCTTCGAGTGGGAGACTTCATCATGATATACAAAGACGATGACAAAAACCGATTTGTCATCCGAGCAAAGAAGGCGGGAGATGATCTAGTTGCTTCTTTGCCACAATTCCACGAGCATATCTCTTCCATTCTGCCAATTCCAGAAGTTGATGACTATGTGTCTCTAATCCCACCACCAGCTGACATCTCTGCCTTTGTGCCACAAGCTGATGAGAATTACGAGATGTTCGATGGGATTTTCAACTCTCTGCCAGAGATACCGGTAGCCAATGTGAGGTACTCAGACTTCTTCGACCCATTCAGTGATTGTATGGACATGTCGAATCCCGGCCTGAACGCCAACAACTCAGCTAACCTGGGAAGTCATTTCCATGATGAGAGGACTGGGCTTTCTTTGTTTCCCAACCCAACGTCTGGGCCTCTGATGTGA

>*TaSPA*(Y09013)

ATGGAGCCCGTGTTCTTCTCACTGGAGGAGGCGATGCCCGAGCCCGACTCTAACCCCTGCCGGACCTCGTCGCCGCCGCTGGAGGCACACATGCTCGTCGCAGGACTCGGAGGAGTGGGCGCCGGCGAGGTCGTCGGCGGGTGCGCGACGAACGAGTGCGCGACAGAATGGTGCTTCCAGAAGTTCGTGGACGAGCCGTGGCTGCTCAACGTCCCCACCGCGCCAGTGGCGAACCCCGAAGCTTCGACGCTTTACCCTAATCCCACGGCCGAGGGGAGCCGCAAGAGGCCGTACGACGTCCATGAGATGGTGGGCCCGGAGGAGGTCATCCCCACGCCGCCTGCGGCGAGCCCGGTGGTGGACCCCGTGGCGTACAACGCGATGCTCAGACGGAAGTTGGACGCGCATCTCGCCGCCGTCGCCATGTTGAGGACCACTCGGGGAATTTGCCCACAAAGCTCCCATGACAATGGAGCATCGCAAAATTCAGATTCCATCCAAGGCTCAGAAAACCACACCGGAGATGTCAGTTTGCATCAACTTAGCTCTTCCTCATTGGAGCCATCACCATCAGATGGTGATATGGAAGGGGAGGCACAAACAATTGGAACTATGCATATTAGTGCAGAGAAAGCGAATAAGAGGAAAGAATCTAACAGGGATTCGGCGAGACGCTCAAGGAGTAGAAAAGCAGCTCATGCGAAGGAACTAGAGGAGCAGGTCTCACTATTAAGAGTCGCAAATAACTCTTTGATGAGACATCTTGCAGATGTAAGTCACAGATACGTCAATATCTCTATTGACAATAGGGTACTAAAGGCAAATGTTGAAACCCTAGAAGCAAAGGTAAAGATGGCCGAGGAAACTATGAAGAGGGTTACATGCACCAACAATTTCCCCCAAGCAATGTCTAGCATATCATCTCTCGGGATTCCTTTCAGTGGCTCCCCATTGAACGGTATCTGTGATAATCCATTGCCAACCCAGAACACCTCACTTAACTACCTCCCTCCCACAACAACAAATTTTGATGTGAACAACAACTACATCCCCGAGCCAGCTCTGGCGTTCCAGATCCAGGATCAAATACCTTCGCTACATATGCAACCTATGTCATGCTTGGATCATCACCCGCAGAGGATGCACATAGGTATTCCTACATCAGCACCTACTCCGCAACGGGAATCTACTACATTGGATTCAACTGAAATAGTCAACATGGTGATGTAG

>*TaPBF*(AJ012284)

ATGGAGGAAGTGTTTTCGTCAAACTCCAAGAGCAAGGCAG GTCAGATGGC GGGGGAGGCGATAGCGGGGGCGGAGAAGAAGCCTCGGCCAAAGCCAGAGCAGAAGGTGGAATGCCCTCGGTGCAAGTCTGGCAACACCAAGTTCTGCTACTACAACAACTATAGTATGTCTCAGCCCCGCTACTTCTGCAAGGCCTGCCGCCGCTACTGGACCCATGGTGGCTCCCTCCGCAACGTCCCCATCGGTGGTGGCTGCCGCAAGCCCAAGCGCTCGGGGACCTCCGACGCCCACAAGCTCGGCGTGGCCTCCTCACCGGAACCCACGACTGTCGTGCCCCCTTCGACCTGCACAGGGATGAACTTTGCGAACGTCCTCCCGACGTTTATGTCTGTTGGTTTTGAGATTCCAAGCAGCCTTTCCCTAACCGCCTTTGGGTCATCATCGTCGTCCAACACGGCGGCGATGATGTCCCCTGGTGGGACGACGTCGTTTCTAGACGTGCTAAGAGGGGGTGCAGGAGGGCTTCTTGATGGCAGCCTCAGTCAGAACAATGGCTACTACTATGGTGGGCCAGCCATTGGATCAGGCAATGGGATGCTGATGACGCCGCCAGCGGTGTCATTTGGCATTCCAGTTCCGATGCAGCAGCATGGCGATCTCGTGGTTGGTGGAAATGGAATAGGTGCCGCAACTGCTTCAATATTTCAAGGGGCCACTAGCGAGGAAGGAGATGACGGCATGGGGGGCGTGATGGGGCTCCAATGGCAACCACAGGTTGGCAATGGTGGAGGTGGTGGTGGTGTATCAGGAGGCGTGCATCACCTCGGGACTGGGAACAATGTGACGATGGGCAACAGCAACATACACAACAACAACAATAACGACAGCGGCGGTGATGACAACAATGGTGGGTCATCGAGGGATTGCTACTGGATCAACAATGGAGGATCAAACCCATGGCAGAGCCTCCTCAACAGCACCTCCCTGATGTAA

>*TaGAMYB*(JF951917)

ATGTACCGGGTGAAGAGCGAGAGCGACTGCGAGATGATGCACCAGGAGGACCAGATGGACTCGCCGGTGGGCGACGACGGCAGCAGCGGCGGAGGGTCGCCTCACAGGGGCGGCGGGCCGCCTCTGAAGAAGGGGCCCTGGACGTCCGCGGAGGACGCCATCCTGGTGGACTACGTGAAGAAGCACGGCGAGGGGAACTGGAACGCGGTGCAGAAGAACACCGGGCTGTTCCGGTGCGGCAAGAGCTGCCGCCTCCGGTGGGCGAACCACCTCAGGCCCAACCTCAAGAAGGGGGCCTTCACCCCCGAGGAGGAGAGGCTCATCATCCAGCTCCACTCCAAGATGGGCAACAAGTGGGCTCGGATGGCCGCTCATTTGCCAGGGCGTACTGATAATGAAATAAAGAATTACTGGAACACTCGAATAAAGAGATGTCAGCGAGCCGGTTTGCCAATATATCCTGCTAGTGTATGCAATCAATCTTCAAATGAAGATCAGCAGGGCTCCAGCGATTTCAACTGCGGCGAGAATCTTTCCAGTGACCTCCTGAATGGAAATGGTCTTTATCTGCCAGATTTTACCTGTGACAATTTCATTGCTAATTCAGAGGCTCTATCTTATGCACCACAGCTTTCAGCTGTTTCAATAAGCAGTTTGCTTGGCCAGAGCTTTGCATCCAAAAACTGCGGCTTCATGGATCAAGTAAACCAAGCAGGGATGCTAAAACAGTCTGACCCTTTACTCCCTGGATTGAGCGACACCATCAATGGCGCGCTCTCCTCGGTCGATCAATTCTCAAATGACTCTGAGAAGCTCAAGCAGGCTCTTGGTTTTGACTATCTCCACGAAGCCAACTCTAGCAGCAAGATTATTGCACCATTTGGGGGTGCGCTTACTGGCAGCCATGCCTTTTTAAATGGCACCTTCTCTACTTCTAGGACCATCAATGGTCCTTTGAAGATGGAGCTCCCTTCACTCCAAGATACCGAATCTGATCCGAATAGCTGGCTCAAGTATACCGTGGCTCCTGCGATGCAGCCTACGGAGTTGGTTGATCCGTACCTGCAGTCCCCGACAGCAACTCCGTCAGTGAAATCGGAGTGTGCTTCGCCGAGGAACAGCGGCCTCTTGGAAGAGCTGCTTCATGAAGCTCAGGGACTAAGATCTGGGAAGAATCAGCAGCTCTCCGTGAGAAGTTCAAGTTCCTCTGTCAGCACGCCGTGTGATACCACGGTGGTTAGCCCGGAGTTTGATCTCTGTCAGGAATATTGGGAAGAACGTCTGAATGAATATGCCCCATTCAGTGGCAATTCACTCACTGGATCCACCGCTCCTATGAGTGCTGCGTCGCCTGATGTTTTTCAGCTCTCCAAAATTTCTCCTGCACAAAGCCCTTCACTGGGATCTGGAGAGCAGGCAATGGAGCCTGCATATGAGCCCGGGGCAGGGGACACTTCGTCTCATCCTGAAAACTTGAGGCCAGACGCGTTCTTCTCCGGGAACACGGCGGACTCGTCCGTCTTCAACAACGCCATAGCCATGCTCCTGGGCAACGACATGAACACGGAGTGCAAGCCTGTTTTCGGCGACGGTATCATGTTTGATACTTCGGTGTGGAGCAACTTGCCTCATGCTTGTCAAATGTCGGAGGAATTCAAATGA
