# Supplementary material for: Functional Characterization of TaFUSCA3, a B3-Superfamily Transcription Factor Gene in the Wheat
Source: Front Plant Sci. 2017 Jun 28;8:1133. doi: 10.3389/fpls.2017.01133 (PMC5487486; doi:10.3389/fpls.2017.01133)
Supplement: Supplementary file 7 [file Table_5.DOCX]

**Supplementary Table S5** The promoter sequences of the seed storage protains in our study.

>At2S3 promoter (At4g27160)

AAACCAAATTAACATAGGGTTTTTATTTAAATAAAAGTTTAACCTTCTTTTTAAAAAATTGTTCATAGTGTCATGTCAGAACAAGAGCTACAAATCACACATAGCATGCATAAGCGGAGCTATGATGAGTGGTATTGTTTTGTTCGTCACTTGTCACTCTTTTCCAACACATAATCCCGACAACAACGTAAGAGCATCTCTCTCTCTCCACACACACTCATGCATGCATGCATTCTTACACGTGATTGCCATGCAAATCTCCTTTCTCACCTATAAATACAAACCAACCCTTCACTACACTCTTCACTCAAACCAAAACAAGAAAACATACACAAATAGCAAAAC

>HMW-1Bx7 promoter (DQ119142)

AACATAGTGTGGTAAATTAAGTGGTACTAACAAAGACATATGCATTGTCAGTTTTTTCTTCTGCCAAACGAAATTTAAGCCTTTGTGTAAACACACATGAGACAACTACAAACAACATCTCTGAAGAATTTAGCCAAATACATTGTAAACTATCTTGGGATATGTGCAAGGTCTGCTGGTATCTCCTTGCATGACGTGATAGTAAAGTAGCTTCTTGAATAAGATTTGAAAAATTCTCCTTGCTGGGTCAATAATTGTTTTTTTCTCTCTCTCAAGGAGTCCTCTCATTCCATTCTTTTAATTCATTATGCTCCCTAATTTTGGAGCTCTCCCATCCAATTGAAATATTCAATTTTAGATTTGGTTCACAATTTTGATCGGGTCGATGGTATCAATCCCGCGCATCCTCTCACCATAAAAAAAAGAGTCAATATATGATATTGTAGCATTAATACATTACATGTAGCCACCGACATGGAAAACGTCCATGCATAAGTATGGTTGATTAATGAAAAACTCATAATCACACTGCATTTTTATAAATAAAAAGAAAATACACTTCATCATCTTCCTCACACACCAAGCCAAATACTCCATCTGTTTCTTTGACCAAGTTCACAACCAAAAACATCAATATCCATAATACTAAATAAATAAAATATGAAAATTCGTTTCATGCTTAATCCAATGATATCGACTTGATATTATGGATATTTATGTATTTCTCTACAAATTTGATCAAACTTTAAAAGGTTTAACTTCTCAAAAAAAAAACTAATACACCTTATATTTTAAAACAGAGAGAGTAGTTTTTTTTTACCCAAACCCCAACTGCCCAGCAAAGCGCGCTCAACTCTTCTAGTCTAAATAACTAGCATCCACTAACACATTTCTCCCGACATGCAAGCACGTCACCTATGAAAATGCCCACCTCAATATGCAACCATGCATAGAAGAAAGCTCACCTCAGCATGCAAACATGCAGCATAATTTCCATTTTACTTGGCTATTTATGTTTGATAAATATTTCACAAATATACAATAATCAAAAACAATAAATTATATGTGTTTTTAGTTTTAGTTCTCATATCCAAATATACATGTTTCATACAACCAAATCTCATTTAAATATATTGTAAAATATTCCGGCAACAACTTGTGGGGGCCTTAAATATATTGTAAAATATTCCGGCAACAACTTGTGGGGTACATCTAGTTACAGTGGAATATTAGTGATGGCGTGACCAAGCGATAAGGCCAACGAGAGAAGAAGTGCGTCGTCTATGGAGGCCAGGGAAAGACAATGGACATGCAAAGAGGTAGGGGCAGGGAAGAAACACTTGGAGATCATAGAAGAACATAAGAGGTTAAACATAGGAGGGCATAATGGACAATTAAATCTACATTAATTGAACTCATTTGGGAAGTAAACAAAATCCATATTCTGGTGTAAATCAAACTATTTGACGCGGATTTACTAAGATCCTATGTTAATTTTAGACATGACTGGCCAAAGGTTTCAGTTAGTTCATTTGTCACGGAAAGGTGTTTTCATAAGTCCAAAACTCTACCAACTTTTTTGCACGTCATAGCATAGATAGATGTTGTGAGTCATTGGATAGATATTGTGAGTCAGCATGGATTTGTGTTGCCTGGAAATCCAACTAAATGACAAGCAACAAAACCTGAAATGGGCTTTAGGAGAGATGGTTTATCAATTTACATGTTCCATGCAGGCTACCTTCCACTACTCGACATGGTTAGAAGTTTTGAGTGCCGCATATTTGCGGAAGCAATGGCACTACTCGACATGGTTAGAAGTTTTGAGTGCCGCATATTTGCGGAAGCAATGGCTAACAGATACATATTCTGCCAAACCCCAAGAAGGATAATCACTCCTCTTAGATAAAAAGAACAGACCAATGTACAAACATCCACACTTCTGCAAACAATACACCAGAACTAGGATTAAGCCCATTACGTGGCTTTAGCAGACCGTCCAAAAATCTGTTTTGCAAGCACCAATTGCTCCTTACTTATCCAGCTTCTTTTGTGTTGGCAAACTGCCCTTTTCCAACCGATTTTGTTCTTCTCACGCTTTCTTCATAGGCTAAACTAACCTCGGCGTGCACACAACCATGTCCTGAACCTTCACCTCGTCCCTATAAAAGCCCATCCAACCTTCACAATCTCATCATCACCCACAACAC CGAGCACCCCAATCTACAGATCAATTCACT GACAGTTCACTGAG

>HMW-1Dx5 promoter (AJ301618)

GCATGCAAATATGCAACATAATTTCCTTTTTACTTGGCTAATTATATTTGATAAATATTTCACAGATATACAATAATCAAACACAATAAATCATATGTGTTTTTAGTTTTAGTTCTCATATCCAAATATACAATAGCTAACCAAATCTCATCGGGAAGTTAGCCATGCCGAGGTAGGTTGTTGCCGGAATGTTTTTAGTTTTAGTTCTCATACAACCAAATCTCATTCAAATATATAAAACATTCCGGCAACAACTTGTGGCGTACATCTAGTTACAAGG GAATATTAGT GATGGCGTGA

GCAAGCGATAAGGCCAAGGAGAGAAGAAGTGCATCGTCTACGGAGGCCAGGGAAAGACAATGGACATGCAGAGAGGCAGGGGCGGGGAAGAAACACTTGGAGATCATAGAAGAAGATAAGAGGTTAAACATAGGAGGAGGATATAATGGACAATTAAATCTGCGTTAGTTGAACTCATTTGGGAAGTAAACAAATTTTCTATTCTGTGTAAACCAAACTATTTGACGCGGATTTTCTCTGAAGATCCTATATTAATTTTAGACATGGTTTGGCTAGTTCATTTGTCGTGAAAAGGTGTTTCCATAAGTCCAAAATTCTACCAACTTTTTTGTATGGCACGTCATAGCATAGATAGATGTTGTGAGTCACTGGATAGATATTGTGAGTCATAGCATGGATTCGTGTTGCTGGAAATCCAACTACATGACAAGCAACAAAACCTGAAATGGGCTTTAGGAGTTAACAATTTACTTGTTCCATGCAGGCTACCTTCCACTACTCGACATGCTTAGAAGCTTTGAGTGGCCGTAGATTTGCAAAAGCAATGGCTAACAGACACATATTCTGCCAAACCCCAAGAAGGATAATCACTTTTCTTAGATAAAAAAGAACAGACCAATATACAAACATCCACACTTCTGCAAACAATACATCAGAACTAGGATTACGCCGATTACGTGGCTTTAGCAGACTGTCCAAAAATCTGTTTTGCAAAGCTCCAATTGCTCCTTGCTTATCCAGCTTCTTTTGTGTTGGCAAACTGCGCTTTTCCAACCGATTTTGTTCTTCTCGCGCTTTCTTCTTAGGCTAAACAAACCTCACCGTGCACG CAGCCATGGTCCTGAACCTTCACCTCGTCCCTATAAAAGCCTAGCCAACC TTCACAATCTTATCATCACCCACAACACCGAGCACCACAAACTAGAGATCAATTCACTGA TAGTCCACC

>HMW-1Bx13 promoter (EF413002)

TGATGTGCCCTTGCTTGATTTCAACATAGTGTGGTAAATTAAGTGGTACTAACAAAGACATATGCATTGTCAGTTTTTTCTTCTGCCAAACGAAATTTAAGCCTTTGTGTAAACACACATGAGACAACTACAAACAACATCTCTGAAGAATTTAGCCAAATACATTGTAAACTATCATGGGCAAGGTCTGCTGGTATCTCCTTGCATGACGTGATAGTAAAGTAGCTTCTTGAATAAGATTTGAAAAATTCTCCTTGCTGGGTCAATAATTGTTTTTTTTCTCTCTCAAGGAGTCCTCTCATTCCATTCTTTTAATTCATTATGCTCCCTAATTTTGGAGCTCTCCCATCCAATTGAAATATTCAATTTTAGATTTGGTTCACAATTTTGATCGGGTCGATGGTATCAATCCTGCGCATCCTCTCACCATAAAAAAAAGAGTCAATATATGATATTGTAGCATTAATACATTACATGTAGCCACCGACATGGAAAACGTCCATGCATAAGTATGGTTGATTAATGAAAAACTCATAATCACACTGCATTTTTATAAATAAAAAGAAAATACACTTCATCATCTTCCTCACACACCAAGCCAAATACTCCATCTGTTTCTTTGACCAAGTTCACAACCAAAAACATCAATATCCATAATACTAAATAAATAAAATATGAAAATTCGTTTCATGCTTAATCCAATGATATCGACTTGATATTATGGATATTTATGTATTTCTCTACAAATTTGATCAAACTTTAAAAGGTTTAACTTCTCAAAAAAAACTAATACACCTTATATTTTAAAACAGAGAGAGTAGTTTTTTTTTACCCAAACCCCAACTGCCCAGCAAAGCGCGCCCAACTCTTCTAGTCTAAATAACTAGCATCCACTAACACATTTCTCCCGACATGCAAGCACGTCACCTATGAAAATGCCCACCTCAATATGCAACCATGCATAGAAGAAAGCTCACCTCAGCATGCAAACATGCAGCATAATTTCCATTTTACTTGGCTATTTATGTTTGATAAATATTTCACAAATATACAATAATCAAAAACAATAAATTATACGTGTTTTTAGTTTTAGTTCTCATATCCAAATATACATGTTTCATACAACCAAATCTCATTTAAATATATTGTAAAATATTCCGGCAACAACTTGTGGGGTACATCTAGTTACAGTGGAATATTAGTGATGGCGTGAGCAAGCGATAAGGCCAACGAGAGAAGAAGTGCGTCGTCTATGGAGGCCAGGGAAAGACAATGGACATGCAAAGAGGTAGGGGCAGGGAAGAAACACTTGGAGATCATAGAAGAACATAAGAGGTTAAACATAGGAGGGCATAATGGACAATTAAATCTACATTAATTGAACTCATTTGGGAAGTAAACAAAATCCATATTCTGGTGTAAATCAAACTATTTGACGCGGATTTACTAAGATCCTATGTTAATTTTAGACATGACTGGCCAAAGGTTTCAGTTAGTTCATTTGTCACGGAAAGGTGTTTTCATAAGTCCAAAACTCTACCAACTTTTTTGCACGTCATAGCATAGATAGATGTTGTGAGTCATTGGATAGATATTGTGAGTCAGCATGGATTTGTGTTGCCTGGAAATCCAACTAAATGACAAGCAACAAAACCTGAAATGGGCTTTAGGAGAGATGGTTTATCAATTTACATGTTCCATGCAGGCTACCTTCCACTACTCGACATGGTTAGAAGTTTTGAGTGCCGCATATTTGCGGAAGCAATGGCTAACAGATACATATTCTGCCAAACCCCAAGAAGGATAATCACTCCTCTTAGATAAAAAGAACAGACCAATGTACAAACATCCACACTTCTGCAAACAATACACCAGAACTAGGATTAAGCCCATTACGTGGCTTTAGCAGACCGTCCAAAAATCTGTTTTGCAAGCACCAATTGCTCCTTACTTATCCAGCTTCTTTTGTGTTGGCAAACTGCCCTTTTCCAACCGATTTTGTTCTTCTCACGCTTTCTTCATAGGCTAAACTAACCTCGGCGTGCACACAACCATGTCCTGAACCTTCACCTCGTCCCTATAAAAGCCCATCCAACCTTCACAATCTCATCATCACCCACAACACCGAGCACCCCAATCTACAGATCAATTCACTGACAGTTCACCGAG
